# Large-scale tumor-associated collagen signatures identify high-risk breast cancer patients

**DOI:** 10.7150/thno.55921

**Published:** 2021-01-01

**Authors:** Gangqin Xi, Wenhui Guo, Deyong Kang, Jianli Ma, Fangmeng Fu, Lida Qiu, Liqin Zheng, Jiajia He, Na Fang, Jianhua Chen, Jingtong Li, Shuangmu Zhuo, Xiaoxia Liao, Haohua Tu, Lianhuang Li, Qingyuan Zhang, Chuan Wang, Stephen A. Boppart, Jianxin Chen

**Affiliations:** 1 Key Laboratory of OptoElectronic Science and Technology for Medicine of Ministry of Education, Fujian Provincial Key Laboratory of Photonics Technology, Fujian Normal University, Fuzhou, China; 2 Breast Surgery Ward, Department of General Surgery, Fujian Medical University Union Hospital, Fuzhou, China; 3 Department of Pathology, Fujian Medical University Union Hospital, Fuzhou, China; 4 Department of Radiation Oncology, Harbin Medical University Cancer Hospital, Harbin, China; 5 College of Physics and Electronic Information Engineering, Minjiang University, Fuzhou, China; 6 Department of Ophthalmology and Optometry, Fujian Medical University, Fuzhou, China; 7 College of Life Science, Fujian Normal University, Fuzhou, China; 8 Department of Medical Oncology, Harbin Medical University Cancer Hospital, Harbin, China; 9 National Center for Supercomputing Applications, University of Illinois at Urbana-Champaign, Urbana, USA; 10 Beckman Institute for Advanced Science and Technology, University of Illinois at Urbana-Champaign, Urbana, USA

**Keywords:** Breast cancer, multiphoton imaging, tumor-associated collagen signatures, disease-free survival

## Abstract

The notion of personalized medicine demands proper prognostic biomarkers to guide the optimal therapy for an invasive breast cancer patient. However, various risk prediction models based on conventional clinicopathological factors and emergent molecular assays have been frequently limited by either a low strength of prognosis or restricted applicability to specific types of patients. Therefore, there is a critical need to develop a strong and general prognosticator.

**Methods:** We observed five large-scale tumor-associated collagen signatures (TACS4-8) obtained by multiphoton microscopy at the invasion front of the breast primary tumor, which contrasted with the three tumor-associated collagen signatures (TACS1-3) discovered by Keely and coworkers at a smaller scale. Highly concordant TACS1-8 classifications were obtained by three independent observers. Using the ridge regression analysis, we obtained a TACS-score for each patient based on the combined TACS1-8 and established a risk prediction model based on the TACS-score. In a blind fashion, consistent retrospective prognosis was obtained from 995 breast cancer patients in both a training cohort (*n*= 431) and an internal validation cohort (*n* = 300) collected from one clinical center, and in an external validation cohort (*n* = 264) collected from a different clinical center.

Results: TACS1-8 model alone competed favorably with all reported models in predicting disease-free survival (AUC: 0.838, [0.800-0.872]; 0.827, [0.779-0.868]; 0.807, [0.754-0.853] in the three cohorts) and stratifying low- and high-risk patients (HR 7.032, [4.869-10.158]; 6.846, [4.370-10.726], 4.423, [2.917-6.708]). The combination of these factors with the TACS-score into a nomogram model further improved the prognosis (AUC: 0.865, [0.829-0.896]; 0.861, [0.816-0.898]; 0.854, [0.805-0.894]; HR 7.882, [5.487-11.323]; 9.176, [5.683-14.816], and 5.548, [3.705-8.307]). The nomogram identified 72 of 357 (~20%) patients with unsuccessful 5-year disease-free survival that might have been undertreated postoperatively.

Conclusions: The risk prediction model based on TACS1-8 considerably outperforms the contextual clinical model and may thus convince pathologists to pursue a TACS-based breast cancer prognosis. Our methodology identifies a significant portion of patients susceptible to undertreatment (high-risk patients), in contrast to the multigene assays that often strive to mitigate overtreatment. The compatibility of our methodology with standard histology using traditional (non-tissue-microarray) formalin-fixed paraffin-embedded (FFPE) tissue sections could simplify subsequent clinical translation.

## Introduction

Breast cancer prognosis after diagnosis plays a central role for patients and oncologists to choose optimally personalized therapies, and thus have motivated the development of a wide variety of survival prognostic biomarkers (prognosticators) 1 and risk prediction models 2. Beyond tumor size and nodal status, the most popular prognosticators are tubule formation, nuclear pleomorphism, and mitotic count that dictate the histological tumor grade 3. The pursuit of similar structural or imaging prognosticators has focused on automatic image analysis and the tumor microenvironment 4. However, the overall trend is to shift from prognosis based on structural tissue features to more functional prognostic features, which, for example, has delivered stronger prognosis for specific types of patients via multigene assays 2, 5. In some cases, structural prognosticators have been developed to “complement” the functional prognosticators of gene expression 6, 7, rather than the other way around. One inadvertent consequence of this trend is an increased emphasis on rare biomolecules such as certain mRNAs and proteins 8, over bulk biomolecules such as lipid, despite the important role of lipid in tumor development 9. Another inadvertent consequence is to focus on cells and intracellular components over extracellular matrix constituents, even though the latter is known to form a dynamic niche in cancer progression 10. This imbalance also holds true in the context of the tumor microenvironment, as emergent prognosticators beyond tumor cells themselves have relied more on the stromal cells 6, 11 or infiltrating immune cells 12 than the extracellular matrix constituents*.*

As one prominent example of this trend, various multigene assays have been compared with computer decision support systems based on conventional clinicopathological factors 13, 14, and have gained prognostic strength for various early-stage patients with young ages (or premenopausal status), few positive lymph nodes, or small tumor sizes 5. However, the restriction to these patients decreases the general applicability of these multigene assays, limiting their usefulness in less developed countries 15 where breast cancer is predominantly diagnosed by signs and symptoms on physical exam (rather than screening) at relatively late stages. In this situation, wherein under-treatment is common and medical budgets are often limited, there is a critical need to develop a strong and general imaging prognosticator compatible with standard histology that outperforms the conventional clinicopathological factors.

Since this type of prognosticator has rarely been clinically validated beyond the infiltrating immune cells in colorectal cancer 12, we aim to test the extracellular structures of interstitial (non-basement-membrane) collagen (bulk biomolecule) that have prominently defied the above trend of prognosticator development and highlighted the frequently underappreciated role of the tumor microenvironment 16-23. Although the corresponding imaging prognosticators have exhibited independent prognostic values over individual clinicopathological factors, they have not been put into a clinical context of a pathological alternative (i.e. multivariate risk prediction model of the clinicopathological factors that makes a computer decision support system available for pathologists) or have not unambiguously demonstrated their differential values over this context, possibly due to their insufficient prognostic strength and general applicability (Table S1).

In this study, we expand the three tumor-associated collagen signatures (TACS1-3) discovered by Keely and coworkers using multiphoton microscopy (MPM) 24, by defining and recognizing five new tumor-associated collagen signatures (TACS4-8) at a larger (~7×) scale and at the invasion front of the primary tumor. We surprisingly find that TACS1-3 (well-established biomarkers) at a scale of 0.4 mm (typical scale for MPM) have significantly lower prognostic strength in comparison to TACS4-8. For the disease-free survival (DFS) prognosis of 995 Chinese patients, the risk prediction model based on TACS1-3 competes poorly with a contextual clinical model developed in our systematic study, while its counterpart based on TACS4-8 (or TACS1-8) considerably outperforms this contextual clinical model and may thus convince pathologists to pursue a TACS-based prognosis. Our methodology of prognosis identifies a significant portion of patients susceptible to undertreatment, in contrast to the multigene assays that often strive to mitigate overtreatment. The compatibility of our methodology with standard histology using traditional (non-tissue-microarray) formalin-fixed paraffin-embedded (FFPE) tissue sections could simplify subsequent clinical translation (Table S1).

## Methods

### Patients

**Figure 1:**
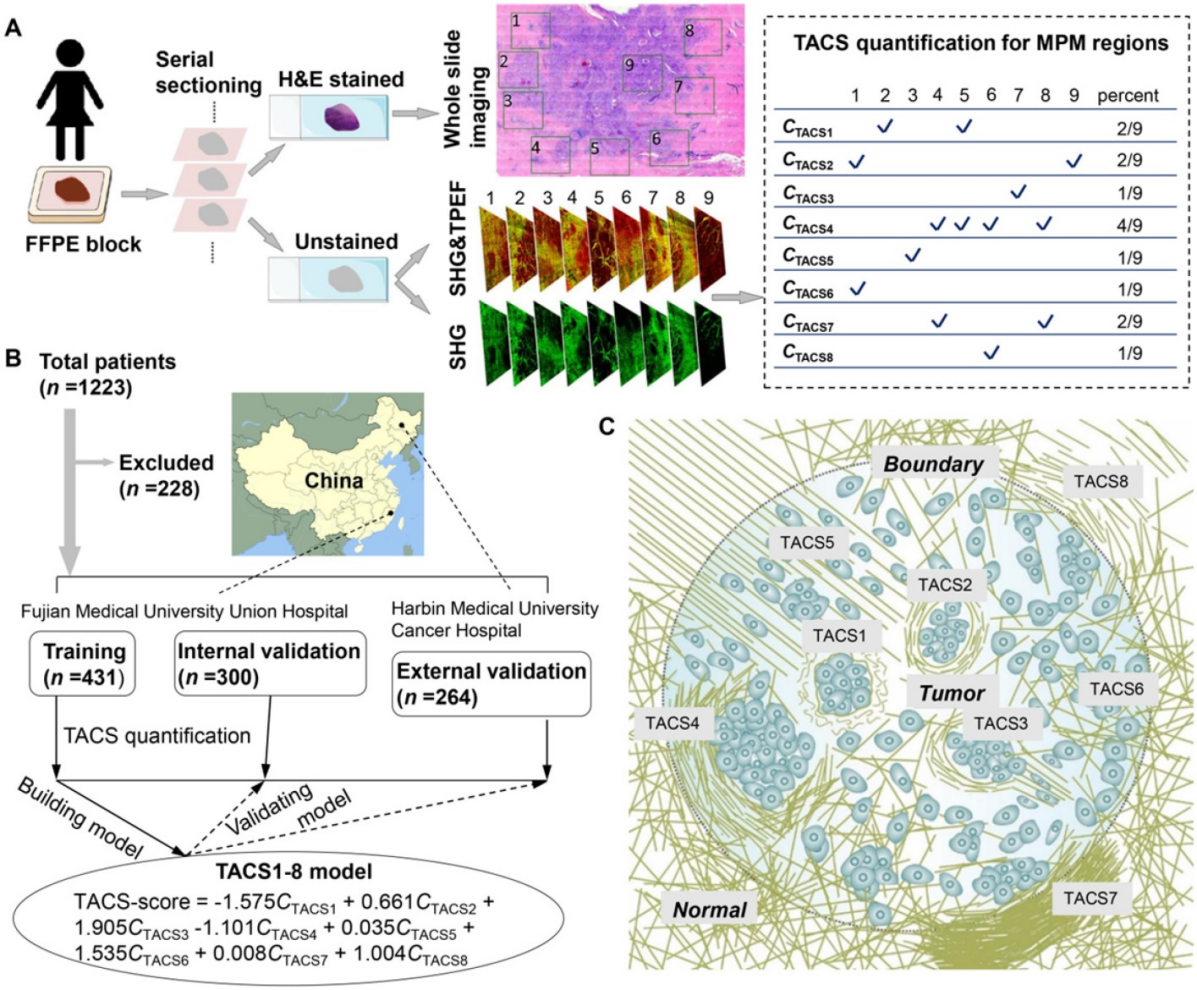
(**A**) Extraction and quantification of TACSs for one exemplary patient among the training, internal validation, and external validation cohorts. For one H&E section of a patient, a total of 9 regions of interest (ROIs) are located either at the invasive front (1-8) or inside the tumor (9). (**B**) Study flowchart to exclude patients with neoadjuvant chemotherapy or radiotherapy, unknown pathological characteristics and follow-up, or damaged and tumor-free sections. The TACS-score is calculated for each patient using the linear combination of TACS percentages weighted by their regression coefficients. (**C**) Illustration of the structural and organizational features of collagen in the TACSs. TACS1-3 are plotted in the tumor center for simplicity but may be present in the invasion front like TACS4-8.

This research used anonymous data for retrospective study and was conducted under a protocol approved by the Institutional Review Boards of Fujian Medical University Union Hospital and Harbin Medical University Cancer Hospital. We collected 1223 FFPE tissue blocks (Figure 1A) from 1223 patients (aged 21-87 years) who underwent surgical resection, in which 228 patients were excluded and 995 patients passed quality control for subsequent analysis (Figure 1B). The inclusion criteria were: (i) patients had pathologically confirmed IBC without distant metastasis and underwent surgical resection; and (ii) clinicopathological characteristics and follow-up information were complete. For the training and internal validation cohorts, 731 samples were obtained between Nov. 2003 and Jun. 2017 from patients treated at the Fujian Medical University Union Hospital (Fuzhou, China). We used computer-generated random numbers to assign 431 of these patients to the training cohort and 300 patients to the internal validation cohort. The external validation cohort comprised 264 patients collected between Jul. 2009 and Dec. 2014 at Harbin Medical University Cancer Hospital (Harbin, China) (Figure 1B).

### Clinicopathologic characteristics and follow-up information

Tumor size was defined as the maximal diameter of the tumor in a resected specimen (T1: the long diameter of the tumor mass is less than 2 cm, T2: the long diameter of the tumor mass is greater than 2 cm and less than or equal to 5 cm; T3: the long diameter of the tumor mass is greater than 5 cm). Nodal status was classified into three categories according to the number of positive lymph nodes (N0: 0 positive lymph node; N1: 1 to 3 positive lymph nodes; N2: more than or equal to 4 positive lymph nodes). Clinical stage (I, II, III) of the tumor was obtained by two surgeons with more than 10 years of clinical experience through reviewing clinical data. Two pathologists with more than 10 years of experience in the diagnosis of breast tumor evaluated the histological grade (G1 to G3) according to the Nottingham histologic grade. The expression of estrogen receptor (ER), progesterone receptor (PR), HER2, and the Ki67 were detected by immunohistochemistry (IHC). Tumors with HER2, IHC staining scores of 0 or 1+ were defined as HER2 negative, while IHC staining scores of 3+ were considered HER2 positive. For IHC staining scores of 2+, molecular detection (*in situ* hybridization (ISH)) was needed to further confirm that the unamplified results of ISH were negative for HER2, and the amplified result of ISH were positive for HER2. According to the detection results of ER, PR, HER2 and Ki67 expression by IHC or ISH, patients were classified into four molecular subtypes as follows: Luminal A: ER positive and/or PR positive, HER2 negative and Ki67 low expression; Luminal B (HER2 negative): ER positive and/or PR positive; HER2 negative and Ki67 high expression; Luminal B (HER2 positive): ER-positive and/or PR positive, and HER2- positive; HER2-enriched: ER negative, PR negative and HER2 positive; Triple-negative: negative for ER, PR and HER2. In this study, age, molecular subtypes, tumor size, nodal status, clinical stage and histological grade were categorical variables. Median DFS follow-up was 70.0 months (IQR 37.0-81.0).

### Multiphoton imaging system

The imaging system was built on a commercially laser scanning microscope platform (LSM 880 Zeiss, Germany) using a mode-locked femtosecond Ti:Sapphire laser that emitted linearly polarized 810 nm excitation light. The backscattered signals from tissue samples were simultaneously obtained via two independent channels. One channel detected second harmonic generation (SHG) signal (green color) between 395 nm and 415 nm while the other channel detected two-photon excitation fluorescence (TPEF) signal (red color) between 428 nm and 695 nm. A Plan-Apochromat ×20 objective (NA = 0.8, Zeiss, Germany) was employed to acquire large field images. Larger scale imaging was enabled by a motorized stage under computer control (ZEN 2.3 SP1 software). The lateral resolution was ~0.8 µm while the imaging field of view was about 0.5×0.5 mm^2^, typically.

### Sample preparation and TACS quantification

**Figure 2:**
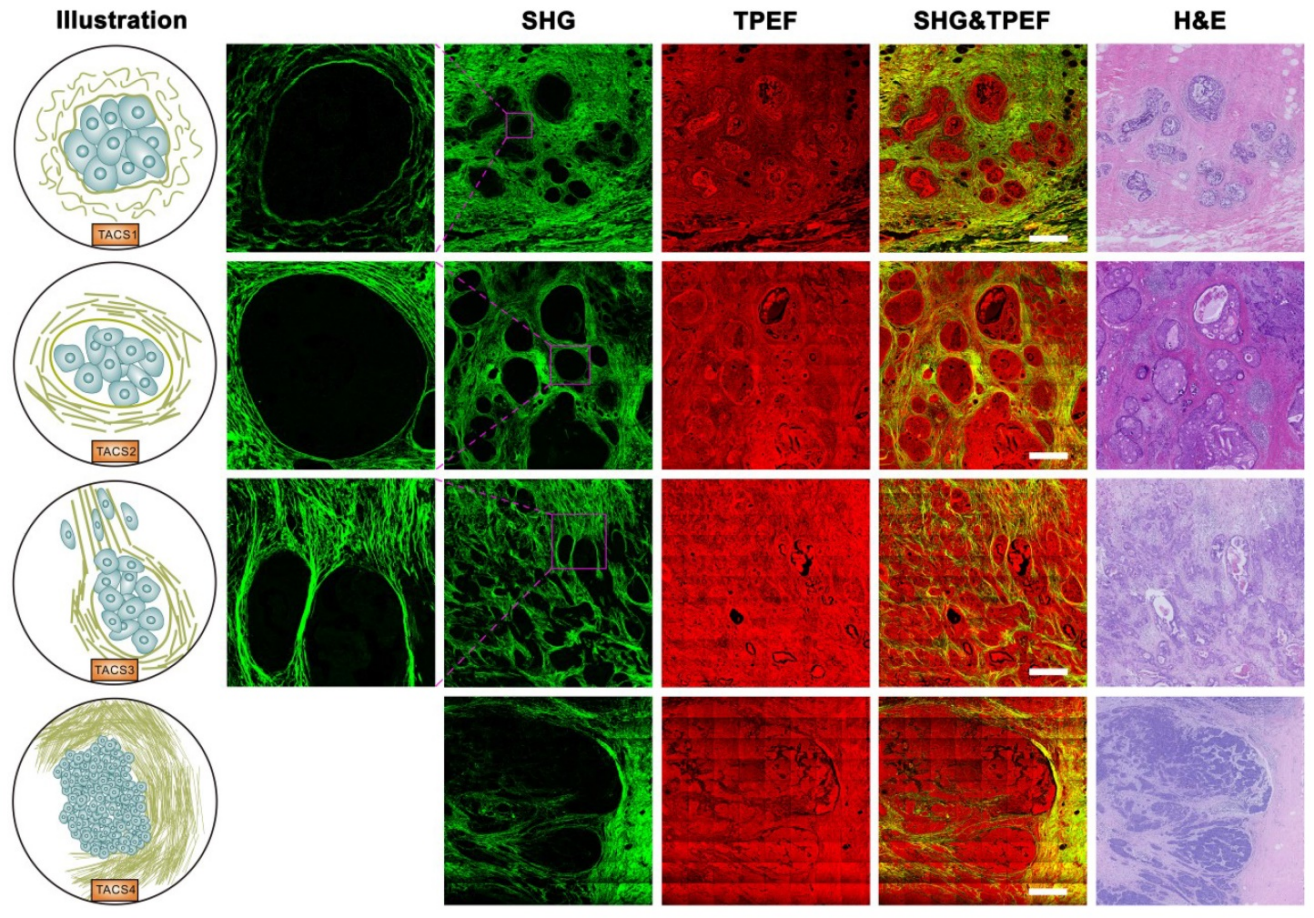
Images of TACS1-3 (or TACS4) at the initiation (or expansion) stage of tumor development. TACS1: curved collagen fibers wrapped around emergent tumor foci; TACS2: collagen fibers stretched due to tumor growth and aligned more parallel to tumor boundary; TACS3: collagen fibers aligned perpendicular to the tumor boundary in a radiation pattern to facilitate tumor cell migration; TACS4: reticular distribution of collagen fibers adjacent to expanding tumor that leads to a clear tumor boundary. Scale bar: 500 μm.

**Figure 3:**
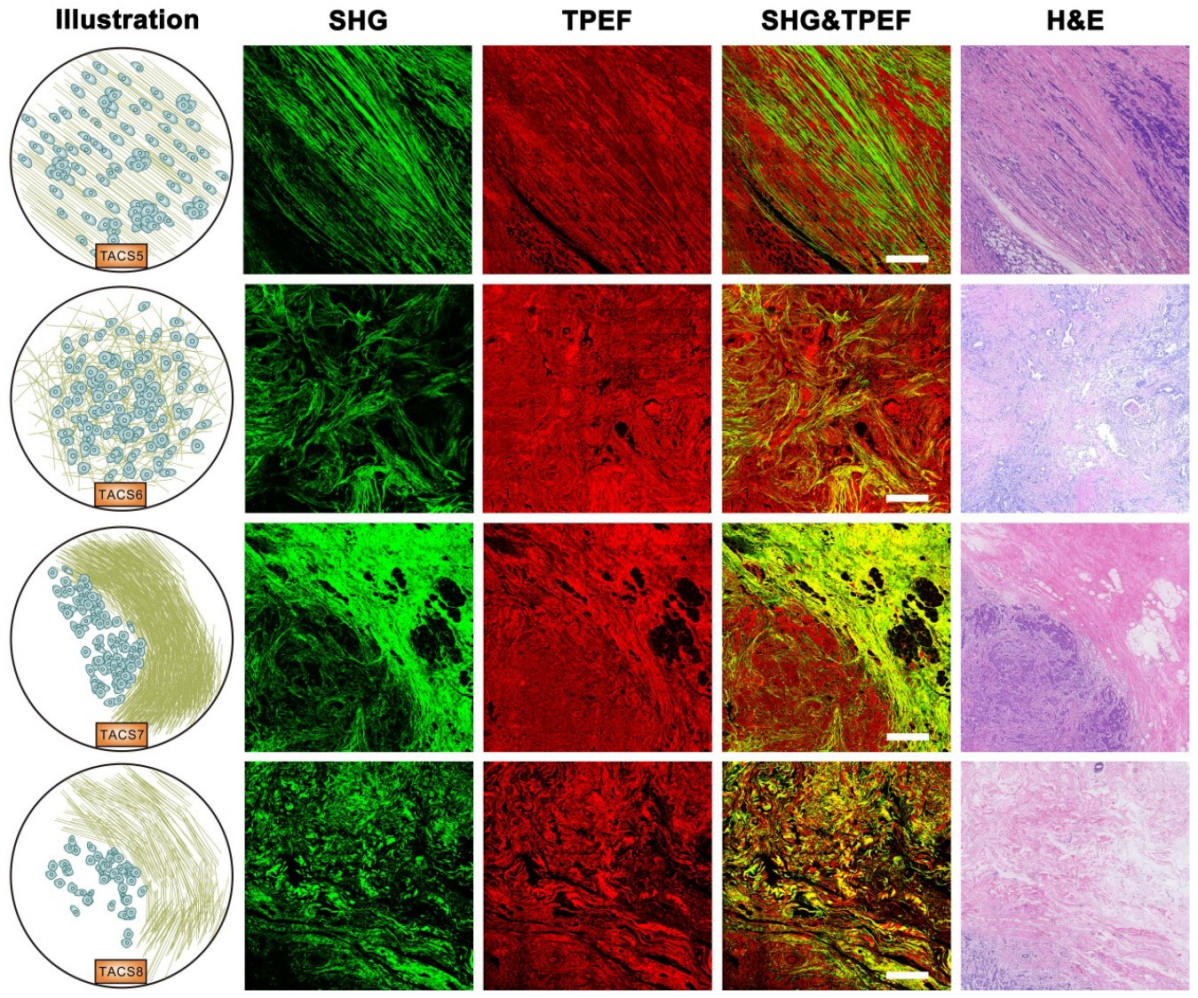
Images of TACS5-8 at the invasion stage of tumor development. TACS5: directionally distributed collagen fibers that enables unidirectional tumor cell migration without a clear tumor boundary; TACS6: chaotically aligned collagen fibers that enables multidirectional tumor cell migration without a clear tumor boundary; TACS7: densely-distributed collagen fibers at the tumor invasion front largely free of tumors cells; TACS8: sparsely-distributed collagen fibers at the tumor invasion front largely free of tumors cells. Scale bar: 500 μm.

For each patient, an archived ~2-cm sized FFPE tissue block containing the invasive front 12, 16, 19 (i.e. the perceived tumor boundary that separates the primary tumor and adjacent normal appearing tissue) was selected for serial sectioning. Two consecutive 5-μm thick serial sections were cut from each paraffin block using a semiautomatic microtome in one pathology laboratory (Figure 1A). One section was used for H&E histology by whole slide imaging, after which a pathologist confirmed the presence of tumor cells and their borders. Throughout the entire tissue section, several (7-20) ~2.8-mm-sized non-overlapping regions of interest (ROI) across the invasive margin and adjacent tumor area were labeled (numbered) in the H&E images (Figure 1A) by two imaging scientists (Lianhuang Li, Gangqin Xi) who were blind to the final pathological outcomes of the patients. ROIs with ductal carcinoma *in situ* (DCIS) were preferably selected inside the tumor, while ROIs were extensively sampled at the invasive front. The other section was deparaffinized by alcohol and xylene. Label-free dual-modal multiphoton microscopy (MPM) simultaneously collecting second harmonic generation (SHG) and two-photon excited fluorescence (TPEF) images 25 was performed for all labeled ROIs on this deparaffinized but unstained section and co-registered with the H&E image (Figures 2-3). This provided an average MPM sampling area of ~60 mm^2^/patient, much larger than that in prior works often employed core needle biopsy (Table S1). Subsequently, the MPM images were visually examined by three independent observers (Jiajia He, Gangqin Xi, Lianhuang Li) who were also blind to the final pathological outcomes. For each ROI, an individual TACS (defined below) was determined to be “present” if at least two reviewers answered “yes” (Figure 1A). The average MPM imaging time on one section (patient) was ~1 h, and typical examination time for one fully trained reviewer to extract TACSs was ~10 min/section.

Intraclass correlation coefficients (ICCs) were used to evaluate the intra-observer and inter-observer discordance of feature determination. To ensure reproducibility, inter-observer correlation coefficients of the TACSs determined by the three observers were calculated. To assess the intra-observer discordance, we randomly selected MPM images of 30 patients for TACS feature extraction. Each panelist performed the extraction for 10 patients and repeated the same steps a week later. The mean intra-observer and inter-observer agreement of the TACS feature extraction among three panelists were 0.921 (95% CI, 0.896-0.945) and 0.857 (95% CI, 0.825-0.888). An ICC value of >0.8 is considered as highly consistent.

### Statistical analysis

Statistical analysis was implemented with R 3.5.2 and IBM SPSS Statistics 24. All statistical tests were two-sided, and a *P*-value of less than 0.05 was considered statistically significant. Univariate and multivariate Cox proportional hazard regression analyses were used to select independent predictors by likelihood ratio test. We used independent predictors to construct the nomogram and generate a comprehensive indicator for estimating disease-free survival (DFS). The performance of nomogram was estimated using discrimination and calibration. The calibration of the nomogram was evaluated by a calibration plot, which was a graphic representation of the relationship between the actual incidence and the predicted probabilities. In a well calibrated model, the predictions should be close to the 45-degree diagonal line. We also constructed receiver operating characteristic (ROC) curves and calculated the areas under the curves (AUCs) to evaluate prognostic accuracy. The ROC curve was used to calculate the optimal cutoff value that was determined by maximizing the Youden index in the training cohort, and then, the same cutoff value was applied to the validation cohorts. The Kaplan-Meier survival curves were further used to estimate the correlation between variables and disease-free survival, and the log-rank test was used to compare differences in the survival.

## Results

### Definition of TACS1-8 and patient-specific quantification

TACS1-3 have been proposed as biomarkers in mouse mammary carcinoma progression 24 and subsequently identified as survival prognosticators for human invasive breast cancer (IBC) 20 and DCIS 21, as well as human ovarian cancer 22 and canine mammary gland carcinoma 23. We examined five new TACS-like (TACS4-8) structures in 431 IBC patients (Figure 1A) intended as the training cohort for our retrospective study (Figure 1B). It should be noted that the differentiation among TACS1-3 (Figure 2) and TACS4-8 (Figures 2-3) occurred at a scale of 0.4 mm and 2.8 mm, respectively. The accumulation of numerous large-scale SHG-TPEF images and co-registered H&E images from the training cohort allowed us to recognize 8 major TACSs (Figure 1C, Figures 2-3), in a manner like how up to 17 histopathological subtypes were identified as biomarkers from H&E images 1. TACS1 and TACS2 resemble those defined in mouse mammary carcinoma 24 except for the lack of dense collagen (TACS1) and the lack of highly straightened (taut) collagen fibers (TACS2). These two TACSs can thus be treated as the human counterparts of the two DCIS-like structures. As reported previously 20, 21, TACS3 reflects a transition from DCIS to IBC due to the breakdown of the basement membrane. Therefore, TACS1-3 can be attributed to the initiation stage of tumor development (Figure 2).

Importantly, TACS4 is defined by a reticular distribution of collagen fibers adjacent to continuously distributed tumor cells, which leads to a clear tumor boundary. While TACS5 is defined by directionally distributed collagen fibers that enable unidirectional tumor cell migration without a clear tumor boundary, TACS6 is defined by chaotically aligned collagen fibers that enable multidirectional tumor cell migration without a clear tumor boundary. Finally, TACS7 is defined by densely distributed collagen fibers at the tumor invasion front largely free of tumors cells, in contrast to TACS8 defined by sparsely distributed collagen fibers at the tumor invasion front largely free of tumors cells. TACS4-8 apparently reflect different tumor-stroma interactions at the invasion stage of tumor development after the DCIS-to-IBC transition (Figures 2-3).

To a large degree, TACS1-3 are located inside the tumor or near the tumor boundary, while TACS4-8 are located at the invasion front near the tumor boundary (Figure 1A, Figure 1C) and can only be easily recognized at a large imaging field-of-view of ~2.8 mm. They might have escaped detection in previous studies that limited imaging field of view to 1 mm in at least one dimension (dictated by the core size of core needle biopsy) or did not differentiate whether the imaging was conducted at the invasion front or tumor center (Table S1) 16-23. For a given patient represented by one FFPE section, multiple TACSs might be present in one ROI and one TACS might exist in multiple ROIs. This complexity was caused by the large size (~2.8 mm) of the ROIs. Regardless of the complexity, TACS information was quantified as an 8-element vector of *C*_TACSi_, which reflected the percentage of TACSi (i = 1, 2, ... or 8) present in all ROIs (Figure 1A).

The quantified TACS information completed the patient-specific quantification in this study that also included age, molecular subtype (Luminal A, Luminal B, HER2-enriched, or Triple-negative), tumor size, nodal status, histological grade, clinical stage, (chemo-, endocrine, radiation, and/or targeted) therapy, and DFS (Tables S2-S3). Recurrence was defined as the regeneration of tumor at the local site or in regional or distant organs after surgical resection of the primary tumor, while DFS was defined as the time from the date of diagnosis (within 3 months from primary tumor surgery) to the date of first recurrence or death 26.

### TACS1-8 as a strong prognosticator

**Figure 4:**
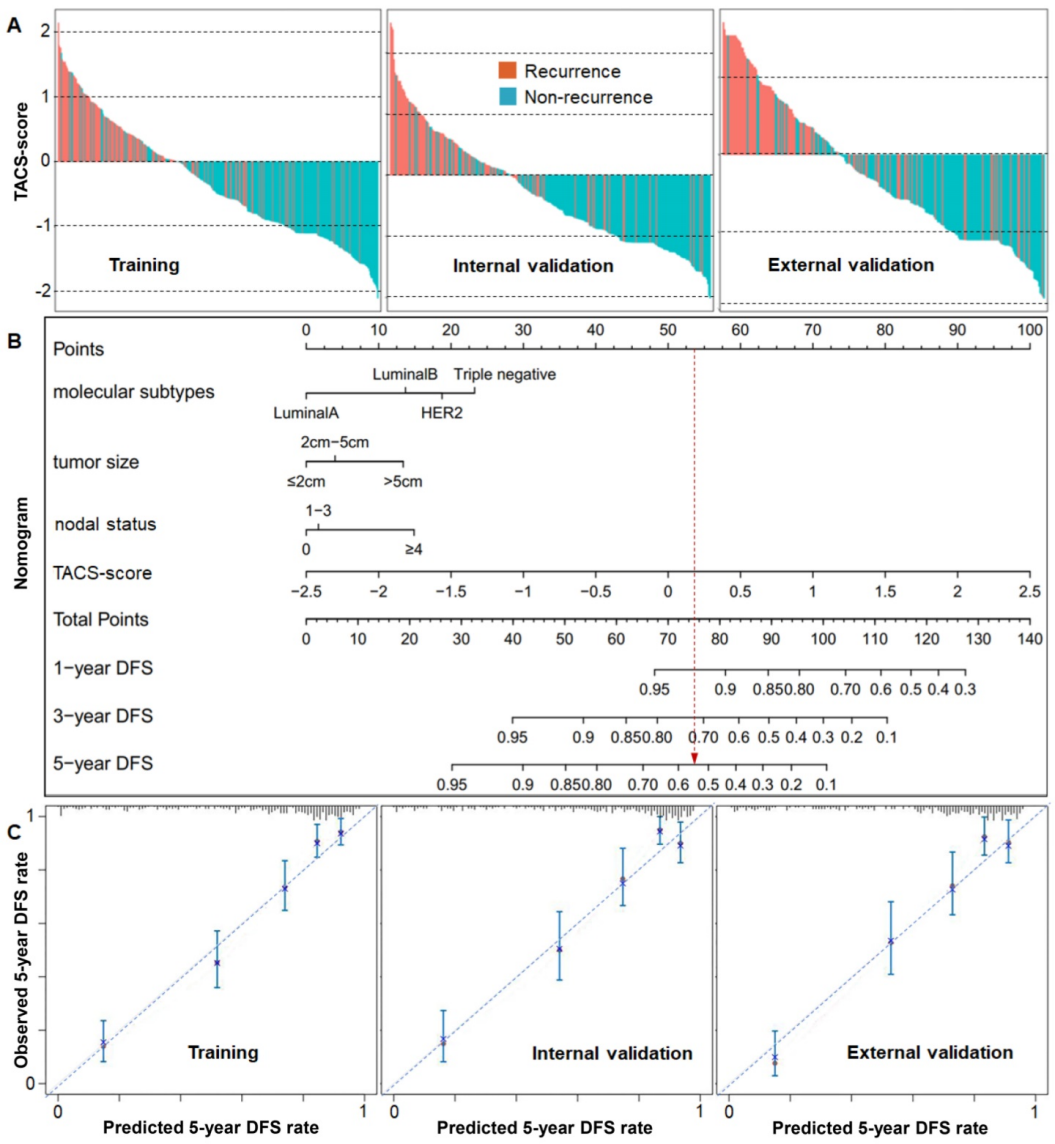
(**A**) Recurrence histograms of TACS-score for three cohorts. (**B**) Nomogram of TACS-score, molecular subtype, tumor size and nodal status derived from the training cohort. (**C**) Calibration curves of the nomogram to predict 5-year DFS rate for three cohorts.

Based on the quantified TACS and DFS data in the training cohorts (Table S2), we used ridge regression with cross validation to retrieve the coefficient of each TACS. The coefficients of all TACSs were fixed in a formula to calculate a patient-specific TACS-score (Figure 1B), which was a manifestation of the 8 TACSs. We then applied this formula to each patient in the training cohort, as well as an internal validation cohort (*n* = 300) and an external validation cohort (*n* = 264) (Figure 1B). In all cohorts, the high and low TACS-scores were consistently associated with high and low recurrence rates, respectively (Figure 4A).

The calculated patient-specific TACS-score, along with age, molecular subtype, tumor size, nodal status, clinical stage, chemotherapy, and endocrine therapy (Table S3), were significantly (*P <* 0.05) associated with DFS in the univariate Cox proportional hazard regression analysis of the training cohort (Table S4, part 3). As expected, older age, Triple-negative subtype, larger tumor size, more positive lymph nodes, higher clinical stage, absence of chemotherapy, absence of endocrine therapy, and higher TACS-score were associated with worse DFS. These prognosticators, except for the endocrine therapy strongly correlated with the molecular subtype (Table S4, part 2), were selected in stepwise building of prognostic model. We used the forward stepwise selection method as the stopping rule to select independent predictors by the likelihood ratio test. In the corresponding multivariate analysis, TACS-score remained as an independent prognostic factor, along with the well-known biomarkers of tumor size, nodal status, and molecular subtype (Table S4, part 1). This was consistent with the increased appreciation of molecular subtype in IBC classification and prognosis 27. The multicollinearity analysis yielded a variance inflation factor of the prognosticators from 1.001 to 1.187 (values far less than 10), indicating that there was no collinearity among these prognosticators.

The risk prediction model containing these independent prognosticators were established from the training cohort and presented as a nomogram, wherein the TACS-score considerably outweighed molecular subtype, nodal status, and tumor size (Figure 4B). Each subgroup of these prognosticators was assigned a point in the corresponding point scale. By summing up the points of all prognosticators in the scale of total points, we could determine the 1-year, 3-year and 5-year DFS rates for a given patient. For example, a patient with a TACS-score of 0.23, nodal status of 0, tumor size of ≤2cm, and molecular subtype of Triple-negative, would have a total point of 75 and predicted 1-year, 3-year and 5-year DFS rates of 92%, 72%, and 54%, respectively (Figure 4B, red line). The calibration curve of the nomogram for 5-year DFS rate showed good agreement between observed and predicted rates in the training cohort (approximation to 45-degree diagonal line is indicative of a well calibrated model), which was retained in the two validation cohorts (Figure 4C). This guaranteed the repeatability and reliability of the established nomogram.

As a reference for TACS-based prognosis, a contextual clinical model was developed based on eight clinicopathological factors (age, molecular subtype, tumor size, nodal status, clinical stage, histological grade, chemotherapy, and radiation therapy) as the computer decision support system available for pathologists. To assess the differential values of TACS4-8 unique to this study, a multivariate model of TACS1-3 was developed in parallel to the TACS1-8 model (TACS-score) discussed above, and would reflect what extent of prognosis could be achieved for these 995 patients from the methodologies of previous works 20-24.

**Figure 5:**
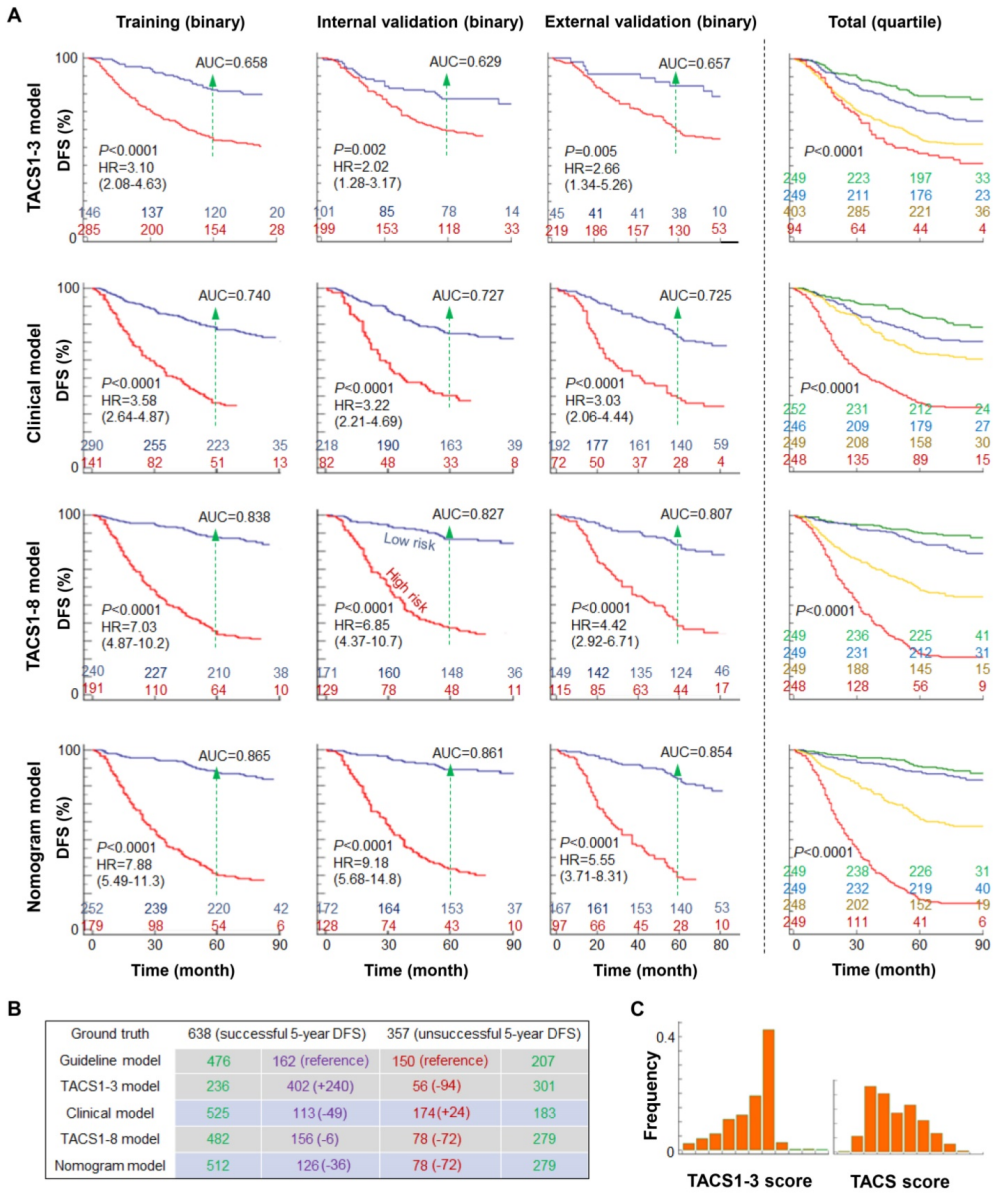
(**A**) Kaplan-Meier curves of DFS according to the TACS1-3 model, clinical model, TACS1-8 model, and nomogram model in four cohorts, with AUC values at the 5-year time point, HR intervals with 95% confidence level, and numbers of patients in two or four risk groups (colored numbers). (**B**) Numbers of likely undertreated (red-highlighted) and overtreated (purple-highlighted) patients according to Chinese treatment guideline and the four models. (**C**) Distribution histogram of TACS1-3 score and TACS-score among 995 patients in the TACS1-3 model and TACS1-8 model, respectively.

The discriminatory accuracy of the clinical model on 5-year DFS rate was reflected by the area under the curve (AUC) of ~0.73 from the receiver operating characteristic (ROC) curve, which expectedly outperformed simpler models based on some individual prognosticators (tumor size, nodal status, and molecular subtype) (Figure 5A, Figure S1A), and approximated the high AUC (0.79-0.81) obtained by including unconventional prognosticcators such as blood test variables, race/ethnicity, cancer detection mode, alcohol consumption, and diagnosis year 26, 28. The TACS1-3 model had low prediction accuracy and led to a lower AUC of ~0.64 for all cohorts, echoing the low prognostic strength of TACS1-3 observed in canine mammary gland carcinoma 23. In contrast, the TACS1-8 model had high prediction accuracy (Figure S1B) and led to a higher AUC of ~0.82 for all cohorts (Figure 5A, Figure S1A). The nomogram model further improved the AUC to ~0.86 and enhanced the discriminatory accuracy (Figure 5A, Figure S1A). As to the corresponding stratification of high- and low-risk patients, the TACS1-3 model, clinical model, TACS1-8 model, and nomogram model delivered an increasing hazard ratio (HR) from ~2 to ~7 (Figure 5A), indicating an increasing ability for risk stratification. Thus, the combined high discriminatory accuracy and super risk stratification ability validated the TACS-score as a strong DFS prognosticator.

### TACS1-8 as a general prognosticator

To demonstrate the general applicability of the TACS-score prognosticator, we combined all cohorts into 995 patients classified by various clinicopathological factors (Table 1). We found that the TACS-score remained statistically significant in all cases. In the Kaplan-Meier analysis, each classified group showed relatively large separation between the low and high TACS risk patients (Figure S2). The high TACS risk of tumor size ≤2cm or 2-5cm had a poorer prognosis than the low TACS risk of tumor size >5cm; the high TACS risk of 0 or 1-3 positive lymph nodes had a poorer prognosis than the low TACS risk of ≥4 positive lymph nodes; the high TACS risk of clinical stage I or II had a poorer prognosis than the low TACS risk of clinical stage III; and the high TACS risk of histological grade G1 or G2 had a poorer prognosis than the low TACS risk of histological grade G3 (Figure S2). These results indicated that the TACS-score risk stratification reclassified a significant portion of patients classified by some well-known clinicopathological factors (i.e. highly complemented these factors), and the TACS-score functioned as a general prognosticator with no restriction to specifically classified patients. The small deficiency in risk assessment of Luminal A patients (with a low AUC of 0.758) was more than compensated by the outstanding ability in risk assessment of HER2-enriched and Triple-negative patients. To the opposite of, yet highly complementary with typical multigene assays, the TACS-score performed better for estrogen receptor-negative patients than for estrogen receptor-positive patients, and attained a higher AUC in highly node-positive (>3) patients than in node-negative (0) patients (Table 1). Interestingly, the TACS-score also performed well for the early stage IBC (Table 1) where the multigene assays have delivered the best value to avoid chemotherapy 2, 5. A larger number of early stage IBC patients will be needed to explore the ability of the TACS-score to mitigate cancer overtreatment.

We also generalized the binary risk stratification of the 995 patients to the corresponding quaternary stratification into equal sized quartiles (Figure 5A, Table S5). In the TACS1-8 model, the Kaplan-Meier curves of the two low-risk quartiles approximated each other but differed significantly from those of the two high-risk quartiles (Figure 5A, right panel). This feature was also observed for the nomogram model but not for the TACS1-3 model or clinical model (Figure 5A, right panel), consistent with the dominant role of TACS-score in the nomogram (Figure 4B).

### Treatment implication

To assess postoperative adjuvant treatment, we used the 5-year prognosis obtained by the clinical, TACS, and nomogram models to evaluate the recurrence risk groups classified by the treatment guideline in China, which is consistent with the 10th St Gallen expert consensus (often considered as the best treatment approach for primary IBC) 29. The execution of this guideline for the 995 patients allowed 626 patients to be stratified as minimum risk (*n* = 32) or moderate risk (*n* = 594) (defined together as low risk) for less aggressive treatment, and 369 patients as high risk for more aggressive treatment (Figure 5B). The guideline caused likely undertreatment to 150 patients and likely overtreatment to 162 patients, which served as the references for the four models: (i) the TACS1-3 model lowered the undertreated patients by 94 at the cost of 240 more overtreated patients, reflecting a worsened performance; (ii) the clinical model lowered the overtreated patients by 49 at the cost of 24 more undertreated patients, reflecting a small improvement; (iii) the TACS1-8 model lowered the undertreated patients by 72 and the overtreated patients by 6, reflecting a larger improvement; and (iv) the nomogram model lowered the undertreated patients by 72 and the overtreated patients by 36, reflecting the best overall improvement (Figure 5B, Figure S3). Thus, our TACS-score-based prognosis showed great potential to complement the standard-of-care treatment guideline in China and improve the choices in tailored adjuvant treatment. In particular, the patients with poor TACS-sore prognosis but low risk under the guideline (72 of 357 patients with unsuccessful 5-year DFS) could have been treated more aggressively. Thus, the TACS-score could be valuable to address the undertreatment in less developed countries, which accounts for 45% (and increasing) of the global burden of breast cancer 15.

### Individual TACS effects

**Figure 6:**
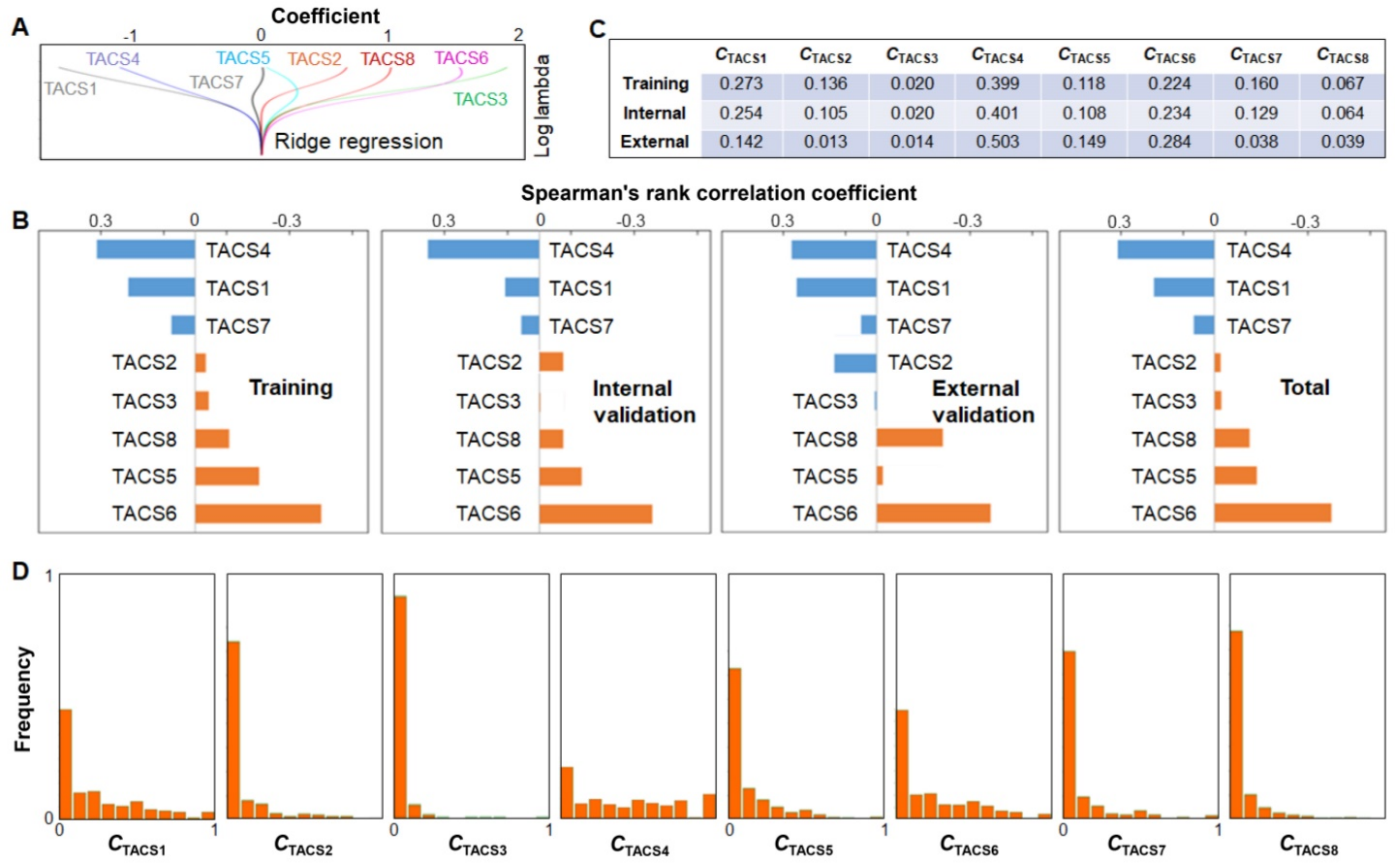
(**A**) Ridge regression of training cohort to attain the coefficients of TACSs in DFS prognosis. (**B**) Correlation analysis between individual TACSs and DFS for four cohorts. (**C**) Average quantified TACS data of one patient in training, internal validation, or external validation cohort. (**D**) Distribution histogram of *C*_TACSi_ among 995 patients in individual TACS models.

To assess the individual effects of the 8 TACSs, we performed similar univariate and multivariate Cox proportional hazard regression analysis as discussed above. While the combinations of independent TACSs exhibited small difference among different cohorts, TACS1, 3, 4, 6, 8 were identified as independent prognosticators for the combined cohort (Table S6). Consistently, the coefficients associated with TACS5 and TACS7 from the ridge regression (training cohort) were small in comparison to those associated with other TACSs (Figure 6A). Whether the TACS prognosticator can be simplified from an 8-structure model to a 6- or 5-structure model will be explored in future studies.

The correlation between individual TACSs and DFS was also assessed by Spearman's rank correlation coefficient. While TACS-specific coefficients exhibited small difference among different cohorts, TACS6, 5, 8 (TACS4, 1, 7) were consistently correlated with poor (good) prognosis (Figure 6B). The significant univariate correlation of TACS5 (TACS7) was obscured in the multivariate analysis (Table S6) and ridge regression (Figure 6A), plausibly by the larger effect of TACS6 (TACS4). In contrast, the small univariate correlation of TACS3, likely due to its low-percentage presence (Figure 6C), emerged as a large coefficient in the (multivariate) ridge regression (Figure 6A). We noted that the external validation cohort (northern Chinese patients, Figure 1B) had rather different percentages of TACS1-8 presence from those of the training or internal validation cohort (southern Chinese patients) (Figure 6C). The consistent results from the two disparate cohorts (Figure 5) reinforced the general applicability of the TACS-score.

We compared the 5-year DFS prognosis using individual TACS models, the clinical model, TACS1-3 model, and TACS1-8 model. We found that the TACS1-8 model showed significantly higher AUC than individual TACS models (Table S7), which was associated with the more even distribution of TACS-score among all 995 patients than a specific *C*_TACSi_ (Figure 5C vs. Figure 6D). Thus, the effects of all TACSs should be comprehensively considered. On the other hand, one model that integrated three independent TACS structures (TACS4, TACS6, and TACS8) outperformed the TACS1-3 model or the clinical model, and approximated the performance of the TACS1-8 (full) model, indicating the disproportional contribution of TACS4-8 over TACS1-3 to the TACS-score (Table S7). In this sense, our study demonstrates the limitation of previous studies based on TACS1-3 alone 20-24.

## Discussion

Stephen Paget's “Seed and Soil” hypothesis suggested that breast cancer metastasis originated from the intricate interaction between tumor cells and their microenvironment 30. Breast tumor microenvironment can regulate tumor progression through extracellular matrix remodeling in tumor-associated stroma 10. From the viewpoint of cellular gene expression, the role of the stroma in regulating breast tumor development and clinical outcome has also been recognized 11, 31. Improved understanding of tumor-associated extracellular matrix and embedded stromal cells at the molecular level has motivated new cancer therapies to target the tumor microenvironment, rather than the tumor cells themselves 32, 33. Parallel attempts have been taken to shift postoperative survival prognosis from tumor cells and intracellular molecules or functions to stromal or extracellular matrix structures 4, 6, 16-23. However, the corresponding tumor microenvironment-based structural prognosticators have not demonstrated compelling strength and general applicability (clinical validity) to justify clinical trials 2, in comparison to various multigene assays based on tumor cells, intracellular biomarker molecules, and biological functions 5. In this study, the TACS-score emerges as a tumor microenvironment-based structural prognosticator with surprising strength of 5-year prognosis in discriminatory accuracy (AUC) and risk stratification ability (HR), which competes favorably with that of the 10-year prognosis by popular multigene assays 34. Unlike the multigene assays, the TACS-score is generally applicable to all IBC subtypes, including the early stage IBC where the multigene assays have delivered the best value (Table 1). The unexpected identification of the TACS-score as a strong and general IBC prognosticator reinforces the central role of the tumor microenvironment in tumor invasion and metastasis. It treats cancer metastasis as a complex tumor-stroma interaction and may therefore produce a survival prognosticator that outperforms the prognosticators based on the tumor cells alone.

The innovations in anatomic pathology (histology) that use structural biomarkers have not kept pace with those of clinical pathology that use functional biomarkers. The invention of MPM in 1990 35 was thought to empower anatomic pathology by introducing new imaging contrasts such as SHG 36. Although MPM has shown promise in *in vivo* optical biopsy to improve cancer diagnosis 37 and interoperative tumor margin detection to improve cancer surgery 38, 39, studies with more direct impact to histology have rarely been clinically validated. One plausible reason is the lack of a precision technology to co-register a virtual imaging section in the intact and often fresh tissue sample (MPM) with one physical section in a FFPE tissue block (histology). Thus, the benefit of additional MPM contrasts over those available from H&E histology and stained immunohistology (IHC) has remained unclear. Our study differs from most clinical MPM studies by imaging a thin FFPE section, an underappreciated sample that is usually avoided to take advantage of MPM in three-dimensional imaging that obviates the need for tissue sectioning and pretreatments. Thus, our MPM-based technology of cancer prognosis inherits some beneficial elements of standard histology: (i) a retrospective study is enabled due to the availability of archived FFPE tissue blocks with well-documented clinicopathological information; (ii) image co-registration is readily achieved (Figure 1A, Figures 2-3) just as H&E-stained histology and stained IHC are correlated; (iii) enabled by this image co-registration, a pathologist can use his/her valuable experience to select local MPM imaging regions near the tumor boundary with a global view of the primary tumor(s) and adjacent normal appearing tissue (Figure 1A), which is important for the TACS-score to attain high prognostic value; and (iv) the associated IHC assays allow the determination of 4 IBC molecular subtypes, which complement the TACS-score to form a better performing nomogram. However, the fundamental difference that distinguishes our technology from standard histology is the replacement of histological grade with the TACS-score as the only structural prognosticator (Table S4). It is striking that the TACS-score overpowers the well-established histological grade 3 in prognostic strength, highlighting the indispensable role of the tumor microenvironment in cancer prognosis. A paradigmatic shift from viewing the discrete cell nuclei stained by hematoxylin to imaging the collagen fibers by SHG (MPM) in our “revamped” histology has yielded a structural prognosticator that can compete with the best functional prognosticators.

Our histology approach that focuses on fibrillar collagen (main component of extracellular matrix) bridges the gap between automated H&E histology 4, 6 that lacks molecular specificity and clinical molecular pathology that lacks structural information. This bulk biomolecule apparently outperforms many biomarkers of rare biomolecules in balancing the sensitivity and specificity of cancer prognosis (Table 1), defying the conventional wisdom that bulk biomolecules are slow or insensitive to respond to subtle cancerous changes. This finding has opened many new fronts of basic and translational research. First, a rational understanding is needed to explain the disparate prognostic values of different TACSs. It may not be surprising that TACS4-8 at invasive or expansion stages and a larger imaging scale are more prognostic than TACS1-3 at tumor initiation stages and relatively small scales. The prognostic differences among TACS4-8 necessitate cell-migration extracellular matrix models that have only been elucidated recently using SHG 40-42. Regardless of the imaging scale, TACS4,7,8 and TACS5,6 are more subtle structures within the TACS framework and may be treated as different variations of TACS2 and TACS3, respectively (Figures 2-3). Second, it will be important to examine whether the TACSs or similar structures of fibrillar collagen can be generalized for risk assessment of other cancer (prostate, lung, skin, liver, etc.) types, considering the broad role of collagen fibers in different organs and epithelial tissues. For one common cancer type (e.g. breast or colorectal cancer), it will be interesting to compare the prognosis by the TACS-score originated from breast cancer and the Immunoscore originated from colorectal cancer 43. Third, a wide variety of other nonlinear intrinsic/label-free contrasts (e.g. TPEF in Figure 3 that reveals cellular information) may synergistically complement the SHG contrast to further improve the prognosis 44, allowing MPM to translate from bench-level laboratorial research to clinical practice. On the other hand, alternative cost-effective and/or high-throughput methods to image collagen fibers, such as the wide-field microscopy with NHS-ester fluorescent labeling 45 or picrosirius red staining 46, may be explored to lower the cost of dedicated TACS extraction. Fourth, this retrospective study provides strong justification for future prospective studies to validate the clinical utility of the TACS prognosticator, which not only demonstrates the ability to mitigate undertreatment in less-developed countries (Figure 5B), but also holds potential to mitigate overtreatment for early stage cancer in well-developed countries (Table 1).

There are several strengths in our study. First, our instrumentation to extract TACSs is highly compatible with standard histology and can be implemented without perturbing the routine histological workflow in future prospective studies (Figure 1A). Alternative technologies using tumor microarrays or gene expression assays are typically less compatible. Second, the standardization of TACS feature extraction is simple after optimizing and fixing the settings of the label-free MPM microscope along with the size and number of imaging regions. Alternative technologies using external staining/labeling in the wet laboratory are typically more resistant to standardization. Third, the FFPE sections allow easy transportation from remote hospitals or other sample collection sites to one central MPM facility and may thus lower the cost of the TACS feature extraction. We also acknowledge some limitations in this study. First, our study is limited to Chinese IBC patients that have relatively low 5-year DFS rate (Table S3) than the IBC patients in most Western countries, even though the TACS-score prognosis exhibits comparable effectiveness in southern and northern China (Figure 1B). Also, our results need to be further validated with longer follow-up (10 years rather than 5 years), to evaluate the long-term effect of the TACSs. Second, the extraction of TACSs necessitates three reviewers and may be prone to interobserver discordance, which may be mitigated by the automatic recognition and quantification of TACSs 47. Third, our prognosis is limited to the DFS that emphasizes quality of life, rather than the overall survival that dictates cancer mortality (which will be further explored to test the validity of the TACSs). Despite these limitations, we believe our study has demonstrated the potential use of TACSs for clinical practice.

## Conclusions

In summary, the current study demonstrates a surprising biomarker (TACS-score) to predict individual disease-free survival rate for breast cancer patients, with great potential to identify high-risk patients (mitigate undertreatment) and expand to other human cancers. The corresponding models have high discriminatory accuracy, superior risk stratification ability, good calibration performance, and broad applicability. This finding strengthens the often-underappreciated role of the tumor microenvironment in (breast) cancer prognosis, introduces innovative features to anatomic pathology, promotes the transformation of multiphoton imaging from laboratory-based research to clinical practice, and enables more informed decisions on adjuvant systemic therapy.

## Supplementary Material

Supplementary figures and tables.

## Figures and Tables

**Table 1:** Prognosis of clinicopathologically classified IBC patients by the TACS1-8 model.

Variable	5-yr DFS	Low risk	HR	95% CI	*P* value	AUC	Sensitivity	Specificity
**Age**								
≤50	369 (66.5%)	329 (59.3%)	5.91	4.32-8.08	<0.0001	0.825	0.747	0.764
>50	269 (61.1%)	231 (52.5%)	6.29	4.42-8.95	<0.0001	0.829	0.819	0.747
**Molecular subtype**								
Luminal A	180 (81.1%)	135 (60.8%)	3.88	2.13-7.05	<0.0001	0.758	0.738	0.689
Luminal B	255 (60.3%)	229 (54.1%)	5.64	4.03-7.91	<0.0001	0.822	0.780	0.757
HER2-enriched	118 (61.1%)	116 (60.1%)	8.35	4.95-14.1	<0.0001	0.871	0.773	0.839
Triple Negative	85 (54.1%)	80 (51.0%)	8.02	4.38-14.7	<0.0001	0.875	0.819	0.788
**Tumor size**								
≤2cm	326 (73.3%)	264 (59.3%)	5.94	4.02-8.77	<0.0001	0.820	0.790	0.736
2-5cm	293 (59.2%)	272 (54.9%)	6.64	4.82-9.14	<0.0001	0.841	0.782	0.778
>5cm	19 (34.5%)	24 (43.6%)	3.62	1.71-7.66	0.001	0.804	0.750	0.789
**Nodal status**								
0	380 (77.7%)	328 (67.1%)	6.07	4.15-8.88	<0.0001	0.828	0.743	0.789
1-3	157 (63.3%)	130 (52.4%)	4.46	2.82-7.05	<0.0001	0.776	0.758	0.688
≥4	101 (39.1%)	102 (39.5%)	5.34	3.57-7.99	<0.0001	0.840	0.822	0.743
**Clinical stage**								
Ⅰ	216 (81.5%)	178 (67.2%)	4.41	2.61-7.47	<0.0001	0.803	0.714	0.759
Ⅱ	317 (68.0%)	278 (59.7%)	6.06	4.25-8.64	<0.0001	0.817	0.758	0.763
Ⅲ	105 (39.8%)	104 (39.4%)	5.37	3.59-8.02	<0.0001	0.833	0.824	0.733
**Histological grade**								
G1	98 (74.2%)	73 (55.3%)	5.48	2.60-11.6	<0.0001	0.780	0.794	0.684
G2	397 (66.3%)	342 (57.1%)	6.45	4.73-8.80	<0.0001	0.828	0.792	0.756
G3	143 (54.2%)	145 (54.9%)	6.09	4.05-9.16	<0.0001	0.853	0.760	0.811
**Estrogen receptor**								
positive	434 (67.8%)	363 (56.7%)	5.06	3.77-6.78	<0.0001	0.798	0.767	0.728
negative	204 (57.5%)	197 (55.5%)	8.47	5.71-12.6	<0.0001	0.878	0.801	0.819
**Young early stage**	120 (83.3%)	102 (70.8%)	4.96	2.40-10.2	<0.0001	0.862	0.708	0.792
**General early stage**	216 (81.5%)	178 (67.2%)	4.41	2.61-7.47	<0.0001	0.803	0.714	0.759

Note: Young early stage invasive breast cancer (Age ≤50, Tumor size ≤2cm, Nodal status 0, Clinical stage I); General early stage invasive breast cancer (Tumor size ≤2cm, Nodal status 0, Clinical stage I).

## Abbreviations

TACS: tumor-associated collagen signatures
IBC: invasive breast cancer
MPM: multiphoton microscopy
SHG: second harmonic generation
TPEF: two-photon excited fluorescence
H&E: hematoxylin and eosin
DFS: disease-free survival
FFPE: formalin-fixed paraffin-embedded
ICCs: Intraclass correlation coefficients
DCIS: ductal carcinoma in situ
AUC: area under the curve
ROC: receiver operating characteristic
HR: hazard ratio
