# Supplementary material for: Large-scale tumor-associated collagen signatures identify high-risk breast cancer patients
Source: Theranostics. 2021 Jan 1;11(7):3229–43. doi: 10.7150/thno.55921 (PMC7847696; doi:10.7150/thno.55921)
Supplement: Supplementary file 1 — Supplementary figures and tables. [file thnov11p3229s1.pdf]

Supplemental Material for:

Large-scale tumor-associated collagen signatures identify high-risk breast cancer patients

Gangqin Xi[1†], Wenhui Guo[2†], Deyong Kang[3†], Jianli Ma[4†], Fangmeng Fu[2], Lida Qiu[1,5], Liqin Zheng[1], Jiajia He[1], Na Fang[1,6], Jianhua Chen[7], Jingtong Li[8], Shuangmu Zhuo[1], Xiaoxia Liao[9], Haohua Tu[10], Lianhuang Li[1✉], Qingyuan Zhang[8✉], Chuan Wang[2✉], Stephen A. Boppart[10✉], and Jianxin Chen[1✉]

1. Key Laboratory of OptoElectronic Science and Technology for Medicine of Ministry of Education, Fujian Provincial Key Laboratory of Photonics Technology, Fujian Normal University, Fuzhou, China
2. Breast Surgery Ward, Department of General Surgery, Fujian Medical University Union Hospital, Fuzhou, China
3. Department of Pathology, Fujian Medical University Union Hospital, Fuzhou, China
4. Department of Radiation Oncology, Harbin Medical University Cancer Hospital, Harbin, China
5. College of Physics and Electronic Information Engineering, Minjiang University, Fuzhou, China
6. Department of Ophthalmology and Optometry, Fujian Medical University, Fuzhou, China
7. College of Life Science, Fujian Normal University, Fuzhou, China
8. Department of Medical Oncology, Harbin Medical University Cancer Hospital, Harbin, China
9. National Center for Supercomputing Applications, University of Illinois at Urbana-Champaign, Urbana, USA
10. Beckman Institute for Advanced Science and Technology, University of Illinois at Urbana-Champaign, Urbana, USA

[†]These authors contributed equally to this work.

✉ Corresponding authors:

Lianhuang Li, E-mail: lhli@fjnu.edu.cn; or Qingyuan Zhang, E-mail: zhma19650210@163.com; or Chuan Wang, E-mail: dr_chuanwang@fjmu.edu.cn; or Stephen A. Boppart, E-mail: boppart@illinois.edu; or Jianxin Chen, E-mail: chenjianxin@fjnu.edu.cn.

Supplementary Figures

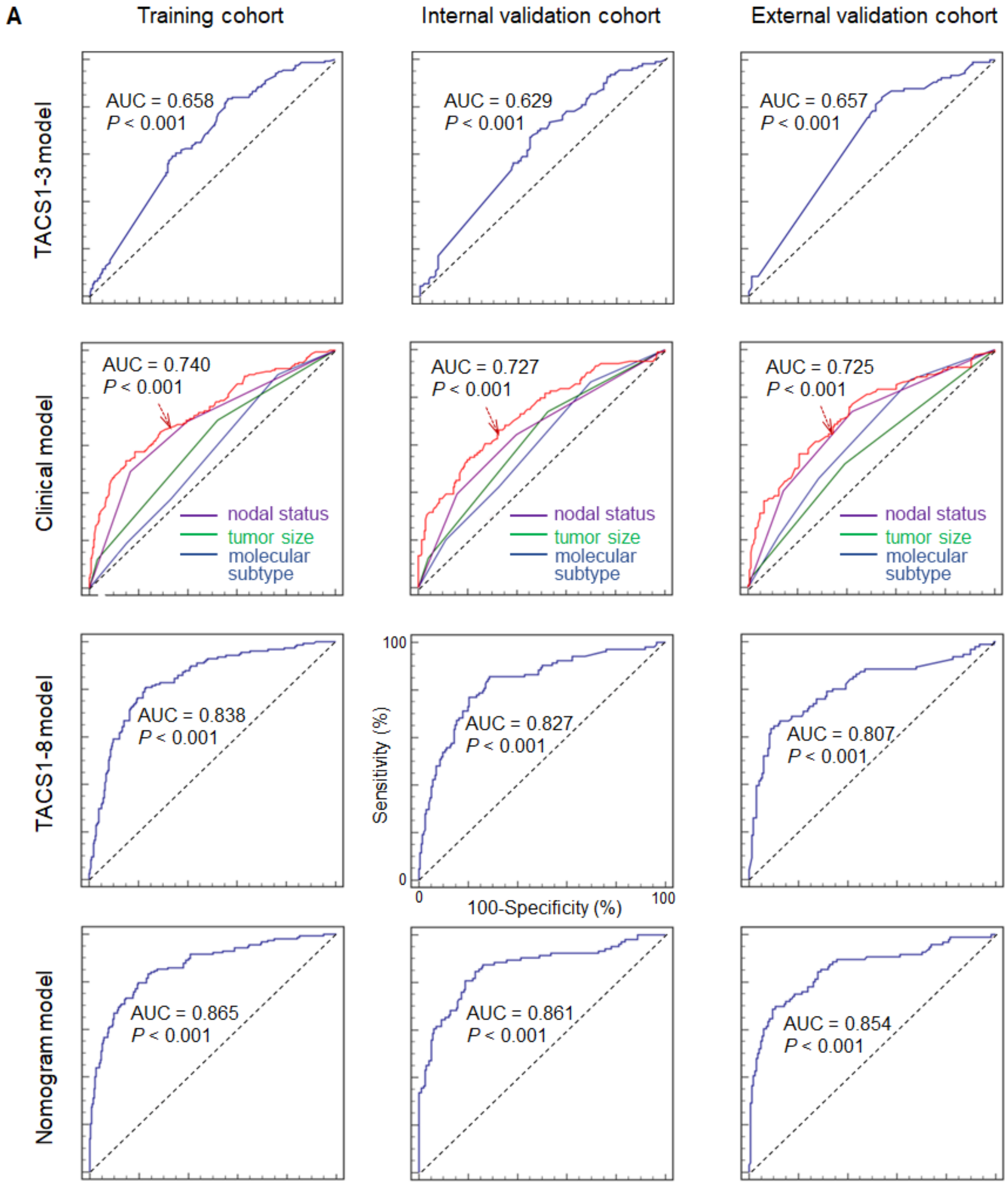

B

TACS1-8 model	Training cohort	Internal validation cohort	External validation cohort
Cutoff score	-0.2487	-0.2487	-0.2487
Sensitivity, %	80.9 (73.9-86.7)	77.9 (68.7-85.4)	74.0 (64.0-82.4)
Specificity, %	77.0 (71.6-81.9)	75.5 (68.9-81.4)	73.8 (66.5-80.3)
Positive predictive value, %	66.8 (61.6-71.7)	62.8 (56.4-68.8)	61.7 (54.9-68.1)
Negative predictive value, %	87.6 (83.5-90.7)	86.5 (81.6-90.3)	83.2 (77.8-87.5)

Figure S1. (**A**) ROC curves of the TACS1-3 model, clinical model and relevant simplified models, TACS1-8 model, and nomogram model to predict 5-year DFS in three cohorts. (**B**) Sensitivity, specificity, positive and negative predictive value (95% confidence level) of the TACS1-8 model to predict 5-year DFS in the three cohorts.

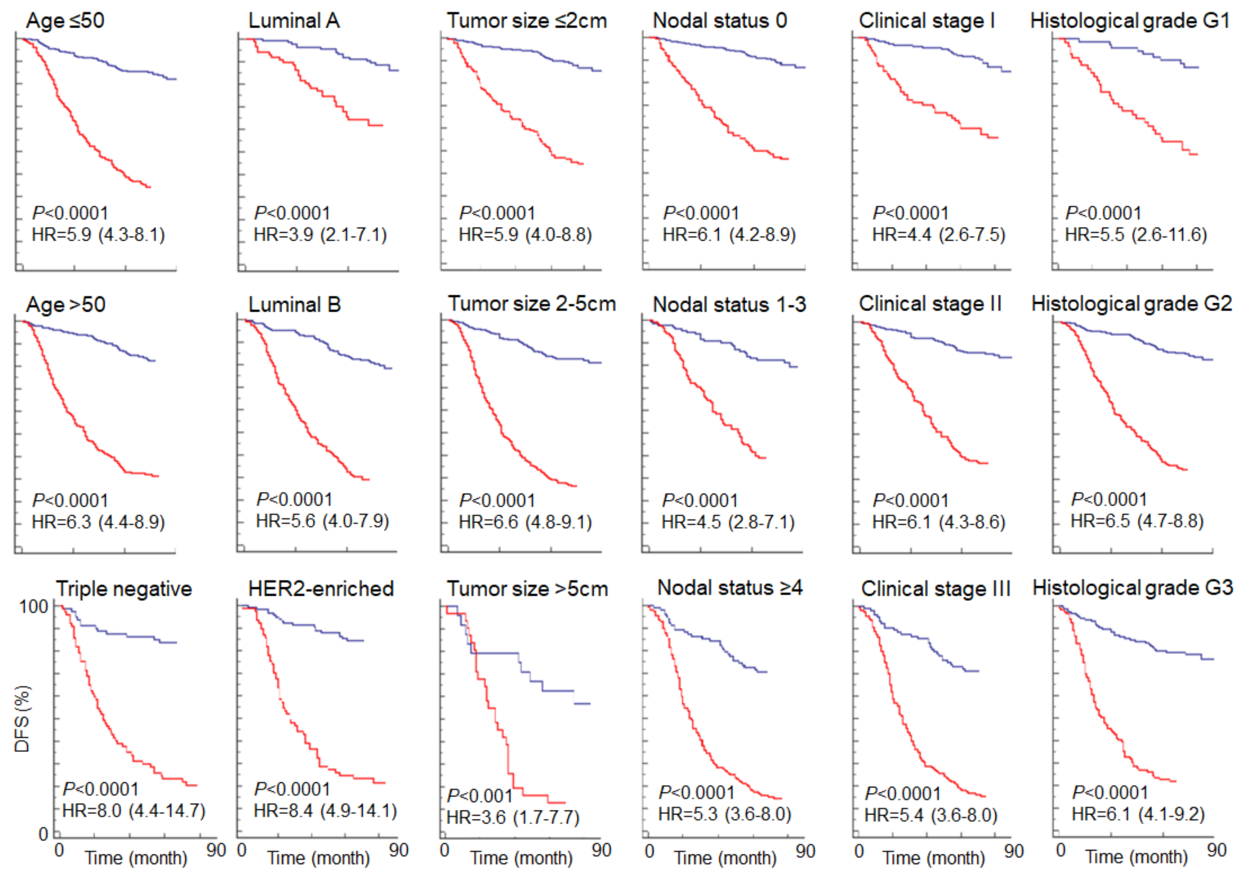

Figure S2. Kaplan-Meier curves of DFS with TACS-score risk stratification for specific patients classified by clinicopathologic factors, with HR shown in an interval with 95% confidence level.

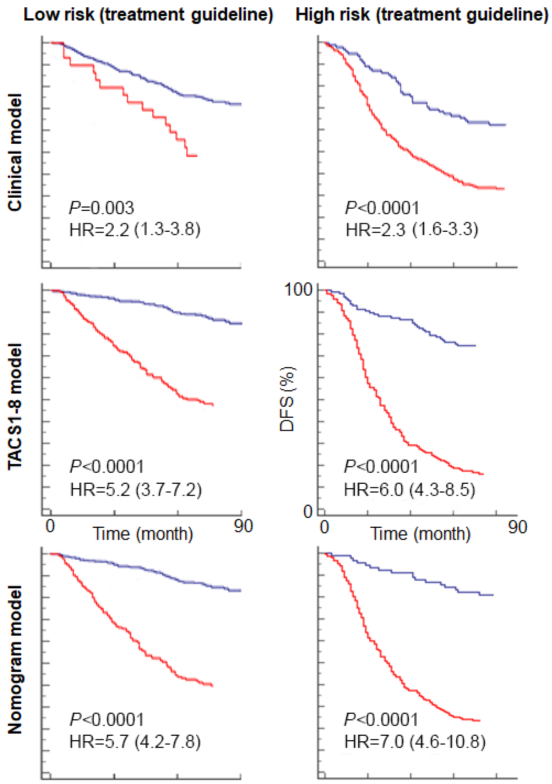

	Low risk in treatment guideline (626)						
	HR	*P* value	AUC	SE	SP	PPV	NPV
Clinical	2.233	0.003	0.623	0.087	0.964	0.433	0.770
TACS	5.169	<0.0001	0.797	0.733	0.752	0.482	0.899
Nomogram	5.743	<0.0001	0.797	0.613	0.857	0.575	0.876

	High risk in treatment guideline (369)						
	HR	*P* value	AUC	SE	SP	PPV	NPV
Clinical	2.328	<0.0001	0.714	0.821	0.407	0.639	0.641
TACS	6.019	<0.0001	0.855	0.816	0.765	0.816	0.765
Nomogram	7.042	<0.0001	0.889	0.903	0.642	0.763	0.839

Figure S3. (Left panel) Kaplan-Meier curves of DFS according to the clinical, TACS1-8, and nomogram models for patients with low risk and high risk under the treatment guideline; (right panel) comparison of predicted 5 year-DFS for patients classified by treatment guideline, where SE - sensitivity, SP - specificity, PPV - positive predictive value, and NPV - negative predictive value.

Supplementary Tables

Table S1 (part 1). Comparison of various studies on collagen structure-based cancer prognosis.

Reference	16	17	18	19	This study
Cancer type	Pancreatic	Prostate	Breast (invasive)	Breast (invasive)	Breast (invasive)
Goal of prognosis	Predict cancer survival	Predict cancer survival	Predict cancer survival	Assess chemotherapy	Predict cancer survival
Origin of samples	1-mm core needle biopsy	0.7-mm core needle biopsy	0.5-mm core needle biopsy	1-mm core needle biopsy	Surgical tissue (no core needle)
FFPE section	Tissue microarray	Tissue microarray	Tissue microarray	Traditional format	Traditional format
Collagen optical imaging method	SHG	Quantitative phase imaging	SHG	SHG	SHG
Number of patients	114 men and women	192 men	221 women	56 women	995 women
Average imaging area/patient	1 mm^2 (~3 cores)	2 mm^2 (5 cores)	0.6 mm^2 (3 cores)	1 mm^2 (3 fields of view)	60 mm^2 (~10 fields of view)
Later resolution	0.8 μm	0.4 μm	0.7 μm	0.7 μm	0.8 μm
Prognosticator(s)	Collagen alignment	Optical anisotropy	SHG F/B ratio	SHG F/B ratio	TACS1-8
Conception of prognosticator(s)	Reported in an early study	Reported in an early study	Reported in an early study	Reported in an early study	TACS4-8 are new biomarkers
Claimed value of cancer prognosis	(Basic prognosis: independently stratify low- and high-risk patients)	Identify high-risk cases for specific patients (Gleason grades 7–10)	Identify low-risk patients susceptible to overtreatment	Correlate chemotherapy response with SHG F/B ratio	Identify high-risk patients susceptible to undertreatment
Co-registered H&E histology	Yes	No	No	Yes	Yes
Differentiation of invasion front from tumor center	Yes (by engaging a pathologist)	Not possible due to tissue microarray	Not possible due to tissue microarray	Yes (by engaging a pathologist)	Yes (by engaging a pathologist)
Context of pathological alternative	Not present as a multivariate risk prediction model	Present as a multivariate risk prediction model	Not present as a multivariate risk prediction model	Not present as a multivariate risk prediction model	Present as a multivariate risk prediction model
Differential value over this context	Not attempted	Not demonstrated	Not attempted	Not attempted	Demonstrated
Prognostic strength insensitive to tumor size	Not demonstrated due to core needle biopsy	Not demonstrated due to core needle biopsy	Not demonstrated due to core needle biopsy	Not demonstrated due to core needle biopsy	Demonstrated (see Table 1, Fig. S2)
Multi-prognosticator nomogram	Not demonstrated	Not demonstrated	Not demonstrated	Not demonstrated	Demonstrated to further improve prognosis
Internal and external validation	Not demonstrated	Not demonstrated	Not demonstrated	Not demonstrated	Demonstrated with high statistical significance
Applicable patients	No restriction discussed	Gleason grades 7–10	Estrogen receptor-positive and lymph node-negative	HER2 positive	General applicability demonstrated
Key limitation of overall prognosis	Relatively low prognostic strength of the prognosticator	Low prognostic strength in comparison to the pathological alternative	Alternative methods of multigene assays good at identifying low-risk patients	Not applicable to triple negative patients and possibly other subgroups	More demanding effort (which can be justified by higher performance and clinical validity)

Table S1 (part 2).

Reference	20	21	22	23	This study
Cancer type	Breast (invasive)	Breast (DCIS)	Ovarian	Breast (invasive)	Breast (invasive)
Goal of prognosis	Predict cancer survival	Predict disease recurrence	Assess degree of malignancy	Predict cancer survival	Predict cancer survival
Origin of samples	1-mm core needle biopsy	Surgical tissue (no core needle)	Surgical tissue (no core needle)	Surgical tissue (no core needle)	Surgical tissue (no core needle)
FFPE section	Tissue microarray	Traditional format	Traditional format	Traditional format	Traditional format
Collagen optical imaging method	SHG	SHG	SHG	SHG	SHG
Number of patients	196 women	227 women	42 women	29 dogs	995 women
Average imaging area/patient	0.8 mm^2 (1 core)	3.5 mm^2 (4.6 fields of view)	Unclear	1.3 mm^2 (5 fields of view)	60 mm^2 (~10 fields of view)
Later resolution	1.2 μm	1.2 μm	0.4 μm	0.7 μm	0.8 μm
Prognosticator(s)	TACS3	TACS3	TACS2, TACS3	TACS1, TACS2, TACS3, and other	TACS1-8
Conception of prognosticator(s)	Reported in an early study	Reported in an early study	Reported in an early study	Reported in an early study	TACS4-8 are new biomarkers
Claimed value of cancer prognosis	(Basic prognosis: independently stratify low- and high-risk patients)	(Basic prognosis: independently stratify low- and high-risk patients)	Correlate degree of malignancy with TACS3	Link survival to collagen density, fiber width, length and straightness	Identify high-risk patients susceptible to undertreatment
Co-registered H&E histology	(Not discussed)	Yes	Yes	(Not discussed)	Yes
Differentiation of invasion front from tumor center	Not possible due to tissue microarray	Not applicable	Not attempted	Invasion front ignored	Yes (by engaging a pathologist)
Context of pathological alternative	Not present as a multivariate risk prediction model	Not present as a multivariate risk prediction model	Not present as a multivariate risk prediction model	Not present as a multivariate risk prediction model	Present as a multivariate risk prediction model
Differential value over this context	Not attempted	Not attempted	Not attempted	Not attempted	Demonstrated
Prognostic strength insensitive to tumor size	Not demonstrated due to core needle biopsy	Not demonstrated	Not demonstrated	Not demonstrated	Demonstrated (see Table 1, Fig. S2)
Multi-prognosticator nomogram	Not demonstrated	Not demonstrated	Not demonstrated	Not demonstrated	Demonstrated to further improve prognosis
Internal and external validation	Not demonstrated	Not demonstrated	Not demonstrated	Not demonstrated	Demonstrated with high statistical significance
Applicable patients	Estrogen receptor-positive and tumor size >1.35 cm	<75-year-old	No restriction discussed	No restriction discussed	General applicability demonstrated
Key limitation of overall prognosis	Low prognostic strength in comparison to estrogen receptor and tumor size	Relatively low prognostic strength of the prognosticator (TACS3)	Indirect relation between degree of malignancy and cancer survival	Relatively low prognostic strength of TACS1, TACS2 and TACS3	More demanding effort (which can be justified by higher performance and clinical validity)

Table S2. Quantified patient-specific data using part of the training cohort as an example.

ID	Age	Subtype	Size	Node	Stage	Grade	CT	ET	RT	TT	TACS1	TACS2	TACS3	TACS4	TACS5	TACS6	TACS7	TACS8	DFS	status
1	≤50	HER2-enriched	2-5cm	0	II	G3	YES	NO	NO	NO	0.000	0.000	0.000	0.000	0.445	0.667	0.111	0.000	21	1
2	≤50	HER2-enriched	>5cm	≥4	III	G2	YES	NO	NO	NO	0.000	0.375	0.000	0.125	0.250	0.500	0.000	0.000	23	1
3	>50	Triple negative	≤2cm	0	I	G2	NO	NO	NO	NO	0.000	0.500	0.000	0.000	0.125	0.625	0.500	0.000	9	1
4	≤50	HER2-enriched	2-5cm	0	II	G2	NO	YES	NO	NO	0.400	1.000	0.000	0.000	0.000	0.000	0.000	0.000	20	1
5	>50	Triple negative	2-5cm	≥4	III	G1	NO	NO	NO	NO	0.000	0.111	0.000	0.000	0.334	0.778	0.111	0.000	30	1
6	>50	Luminal B	>5cm	≥4	III	G2	YES	YES	YES	NO	0.000	0.000	0.000	0.000	0.334	0.778	0.000	0.000	18	1
7	≤50	HER2-enriched	>5cm	≥4	III	G3	YES	NO	YES	NO	0.500	0.625	0.000	0.500	0.000	0.000	0.625	0.000	15	1
8	>50	Triple negative	≤2cm	1-3	II	G2	YES	NO	NO	NO	0.714	0.286	0.143	0.000	0.000	0.714	0.000	0.000	12	1
9	≤50	HER2-enriched	≤2cm	0	I	G3	YES	YES	YES	NO	0.125	0.500	0.000	0.500	0.000	0.500	0.375	0.000	15	1
10	>50	Luminal B	2-5cm	≥4	III	G3	YES	YES	YES	NO	0.125	0.125	0.000	0.375	0.000	0.500	0.375	0.000	12	1
⋮	⋮	⋮	⋮	⋮	⋮	⋮	⋮	⋮	⋮	⋮	⋮	⋮	⋮	⋮	⋮	⋮	⋮	⋮	⋮	⋮
422	>50	HER2-enriched	≤2cm	0	I	G1	NO	NO	NO	NO	0.500	0.333	0.083	0.083	0.000	0.250	0.167	0.000	108	0
423	>50	HER2-enriched	≤2cm	0	I	G3	YES	NO	NO	NO	0.333	0.000	0.000	0.778	0.000	0.000	0.000	0.000	106	0
424	>50	HER2-enriched	2-5cm	0	II	G2	YES	NO	NO	YES	0.000	0.000	0.000	1.000	0.000	0.000	0.250	0.000	100	0
425	≤50	Triple negative	≤2cm	≥4	III	G2	YES	NO	NO	NO	0.000	0.000	0.000	1.000	0.000	0.000	0.000	0.000	137	0
426	≤50	Triple negative	≤2cm	≥4	III	G3	NO	YES	YES	NO	0.000	0.000	0.000	0.600	0.000	0.600	0.000	0.000	135	0
427	≤50	Triple negative	2-5cm	1-3	II	G2	YES	NO	NO	NO	0.000	0.000	0.000	1.000	0.000	0.000	0.667	0.000	113	0
428	≤50	Luminal B	2-5cm	1-3	II	G2	YES	YES	YES	YES	0.167	0.500	0.167	1.000	0.000	0.000	0.333	0.000	79	1
429	≤50	Luminal B	≤2cm	0	I	G1	NO	YES	NO	NO	0.000	0.200	0.000	1.000	0.000	0.000	0.600	0.000	79	0
430	≤50	Luminal B	≤2cm	0	I	G3	YES	YES	NO	NO	0.143	0.143	0.000	1.000	0.000	0.000	0.571	0.000	78	0
431	≤50	HER2-enriched	2-5cm	0	II	G3	YES	NO	NO	NO	0.000	0.000	0.000	1.000	0.000	0.000	0.125	0.000	77	0

Note: ID - identification number; subtype - molecular subtype; size - tumor size; node – nodal status; stage - clinical stage; grade - histological grade; CT – chemotherapy; ET - endocrine therapy; RT - radiation therapy; TT - targeted therapy; DFS - disease-free survival; status: 1 - observed recurrence/death in follow-up, 0 - without observed recurrence/death in follow-up.

Table S3. Baseline characteristics of patients in the three cohorts.

Characteristics	Fuzhou training cohort (431)	Fuzhou internal validation cohort (300)	Harbin external validation cohort (264)	Total (995)
**Age**				
≤50	240 (55.7%)	174 (58%)	141 (53.4%)	555 (55.8%)
>50	191 (44.3%)	126 (42%)	123 (46.6%)	440 (44.2%)
**Molecular subtype**				
Luminal A	80 (18.6%)	73 (24.3%)	69 (26.1%)	222 (22.3%)
Luminal B	201 (46.6%)	119 (39.7%)	103 (39.0%)	423 (42.5%)
HER2-enriched	78 (18.1%)	65 (21.7%)	50 (18.9%)	193 (19.4%)
Triple-negative	72 (16.7%)	43 (14.3%)	42 (15.9%)	157 (15.8%)
**Tumor size**				
≤2cm	177 (41.1%)	120 (40.0%)	148 (56.1%)	445 (44.7%)
2-5cm	226 (52.4%)	159 (53.0%)	110 (41.7%)	495 (49.8%)
>5cm	28 (6.5%)	21 (7.0%)	6 (2.2%)	55 (5.5%)
**Nodal status**				
0	212 (49.2%)	155 (51.7%)	122 (46.2%)	489 (49.1%)
1-3	96 (22.3%)	73 (24.3%)	79 (29.9%)	248 (24.9%)
≥4	123 (28.5%)	72 (24.0%)	63 (23.9%)	258 (25.9%)
**Clinical stage**				
Ⅰ	112 (26.0%)	76 (25.3%)	77 (29.2%)	265 (26.6%)
Ⅱ	197 (45.7%)	147 (49.0%)	122 (46.2%)	466 (46.9%)
Ⅲ	122 (28.3%)	77 (25.7%)	65 (24.6%)	264 (26.5%)
**Histological grade**				
G1	71 (16.5%)	50 (16.7%)	11 (4.2%)	132 (13.3%)
G2	226 (52.4%)	162 (54.0%)	211 (79.9%)	599 (60.2%)
G3	134 (31.1%)	88 (29.3%)	42 (15.9%)	264 (26.5%)
**Chemotherapy**				
No	39 (9%)	23 (7.7%)	27 (10.2%)	89 (8.9%)
Yes	392 (91%)	277 (92.3%)	237 (89.8%)	906 (91.1%)
**Endocrine Therapy**				
No	168 (39%)	117 (39.0%)	141 (53.4%)	426 (42.8%)
Yes	263 (61%)	183 (61.0%)	123 (46.6%)	569 (57.2%)
**Radiation Therapy**				
No	292 (67.7%)	199 (66.3%)	204 (77.3%)	695 (69.8%)
Yes	139 (32.3%)	101 (33.7%)	60 (22.7%)	300 (30.2%)
**Targeted Therapy**				
No	404 (93.7%)	280 (93.3%)	239 (90.5%)	923 (92.8%)
Yes	27 (6.3%)	20 (6.7%)	25 (9.5%)	72 (7.2%)
**5-yr DFS rate**	274 (63.6%)	196 (65.3%)	168 (63.6%)	638 (64.1%)

Table S4 (part 1)**.** Univariate and multivariate Cox proportional hazards regression analysis of the association of variables with DFS in the training cohort (only independent prognosticators included in multivariate analysis).

Variable	Univariate analysis				Multivariate analysis			
	HR	(95%CI)		*P* Value	HR	(95%CI)		*P* Value
**Age**								
≤50	Reference							
>50	1.402	1.036	1.897	0.029	NA			NA
**Molecular subtype**								
Luminal A	Reference							
Luminal B	2.336	1.390	3.925	0.001	2.048	1.202	3.487	0.008
HER2-enriched	2.202	1.218	3.981	0.009	2.654	1.452	4.850	0.002
Triple-negative	2.607	1.442	4.711	0.002	3.353	1.842	6.104	<0.0001
**Tumor size**								
≤2cm	Reference							
2-5cm	1.684	1.198	2.368	0.003	1.232	0.871	1.744	0.238
≥5cm	3.666	2.177	6.172	<0.0001	1.993	1.153	3.446	0.014
**Nodal status**								
0	Reference							
1-3	1.570	1.028	2.398	0.037	1.092	0.707	1.687	0.692
≥4	3.597	2.535	5.102	<0.0001	2.168	1.483	3.169	<0.0001
**Clinical stage**								
Ⅰ	Reference							
Ⅱ	1.902	1.177	3.075	0.009	NA			NA
Ⅲ	4.486	2.789	7.216	<0.0001	NA			NA
**Histological grade**								
G1	Reference							
G2	1.153	0.732	1.814	0.540	NA			NA
G3	1.557	0.969	2.499	0.067	NA			NA
**Chemotherapy**								
Yes	Reference							
No	1.626	1.029	2.570	0.037	NA			NA
**Endocrine Therapy**								
Yes	Reference							
No	1.602	1.183	2.170	0.002	NA			NA
**Radiation Therapy**								
Yes	Reference							
No	0.834	0.608	1.145	0.262	NA			NA
**Targeted Therapy**								
Yes	Reference							
No	1.726	0.810	3.679	0.157	NA			NA
**TACS-score**	2.889	2.434	3.429	<0.0001	2.836	2.359	3.410	<0.0001

Table S4 (part 2)**.** Univariate and multivariate Cox proportional hazards regression analysis of the association of variables with DFS in the training cohort (all prognosticators except endocrine therapy and targeted therapy included in multivariate analysis).

Variable	Univariate analysis				Multivariate analysis			
	HR	(95%CI)		*P* Value	HR	(95%CI)		*P* Value
**Age**								
>50 vs ≤50	1.402	1.036	1.897	0.029	0.955	0.688	1.326	0.783
**Molecular subtype**								
Luminal B vs Luminal A	2.336	1.390	3.925	0.001	1.957	1.124	3.409	0.018
HER2-enriched vs Luminal A	2.202	1.218	3.981	0.009	2.418	1.293	4.522	0.006
Triple-negative vs Luminal A	2.607	1.442	4.711	0.002	2.858	1.496	5.463	0.001
**Tumor size**								
2-5cm vs ≤2cm	1.684	1.198	2.368	0.003	1.251	0.801	1.953	0.324
≥5cm vs ≤2cm	3.666	2.177	6.172	1.0E-06	2.193	1.189	4.044	0.012
**Nodal status**								
1-3 vs 0	1.570	1.028	2.398	0.037	1.091	0.651	1.828	0.741
≥4 vs 0	3.597	2.535	5.102	7.3E-13	2.929	1.101	7.795	0.031
**Clinical stage**								
Ⅱvs Ⅰ	1.902	1.177	3.075	0.009	0.949	0.465	1.937	0.885
Ⅲvs Ⅰ	4.486	2.789	7.216	6.1E-10	0.711	0.222	2.279	0.566
**Histological grade**								
G2 vs G1	1.153	0.732	1.814	0.540	0.925	0.567	1.508	0.754
G3 vs G1	1.557	0.969	2.499	0.067	1.157	0.681	1.967	0.590
**Chemotherapy**								
No vs Yes	1.626	1.029	2.570	0.037	1.310	0.787	2.181	0.299
**Radiation Therapy**								
No vs Yes	0.834	0.608	1.145	0.262	1.058	0.732	1.529	0.766
**TACS-score**	2.889	2.434	3.429	6.6E-34	2.799	2.313	3.386	3.4E-26

Table S4 (part 3)**.** Univariate and multivariate Cox proportional hazards regression analysis of the association of variables with DFS in the training cohort (all prognosticators included in multivariate analysis).

Variable	Univariate analysis				Multivariate analysis			
	HR	(95%CI)		*P* Value	HR	(95%CI)		*P* Value
**Age**								
>50 vs ≤50	1.402	1.036	1.897	0.029	0.961	0.692	1.335	0.812
**Molecular subtype**								
Luminal B vs Luminal A	2.336	1.390	3.925	0.001	2.011	1.151	3.514	0.014
HER2-enriched vs Luminal A	2.202	1.218	3.981	0.009	1.371	0.657	2.864	0.401
Triple-negative vs Luminal A	2.607	1.442	4.711	0.002	1.315	0.624	2.773	0.472
**Tumor size**								
2-5cm vs ≤2cm	1.684	1.198	2.368	0.003	1.180	0.753	1.850	0.470
≥5cm vs ≤2cm	3.666	2.177	6.172	1.03-06	2.237	1.203	4.159	0.011
**Nodal status**								
1-3 vs 0	1.570	1.028	2.398	0.037	0.996	0.590	1.680	0.987
≥4 vs 0	3.597	2.535	5.102	7.3E-13	2.860	1.049	7.799	0.040
**Clinical stage**								
Ⅱvs Ⅰ	1.902	1.177	3.075	0.009	1.080	0.522	2.233	0.836
Ⅲvs Ⅰ	4.486	2.789	7.216	6.1E-10	0.730	0.218	2.436	0.608
**Histological grade**								
G2 vs G1	1.153	0.732	1.814	0.540	0.883	0.543	1.435	0.615
G3 vs G1	1.557	0.969	2.499	0.067	1.240	0.729	2.109	0.427
**Chemotherapy**								
No vs Yes	1.626	1.029	2.570	0.037	1.146	0.686	1.915	0.602
**Endocrine Therapy**								
No vs Yes	1.602	1.183	2.170	0.002	2.712	1.669	4.406	5.6E-05
**Radiation Therapy**								
No vs Yes	0.834	0.608	1.145	0.262	0.867	0.592	1.269	0.462
**Targeted Therapy**								
No vs Yes	1.726	0.810	3.679	0.157	2.138	0.952	4.801	0.066
**TACS-score**	2.889	2.434	3.429	6.6E-34	2.927	2.414	3.548	7.8E-28

Table S5. Hazard ratios (HRs) of DFS according to quaternary risk stratification of 995 breast cancer patients by four models.

Models	Quartile 1	Quartile 2	Quartile 3	Quartile 4	Test for trend
**TACS1-3 model**					
HRs (95% CI)	1.0	1.64 (1.17-2.31)	2.68 (1.98-3.63)	3.51 (2.41-5.11)	*P*=5.5E-13
quartile effect	Reference	*P*=4.6E-03	*P*=1.6E-10	*P*=5.8E-11	
**Clinical model**					
HRs (95% CI)	1.0	1.56 (1.09-2.23)	2.20 (1.57-3.09)	5.20 (3.79-7.13)	*P*=2.9E-31
quartile effect	Reference	*P*=1.6E-02	*P*=5.4E-06	*P*=1.2E-24	
**TACS1-8 model**					
HRs (95% CI)	1.0	1.79 (1.13-2.85)	5.04 (3.33-7.62)	13.1 (8.76-19.4)	*P*=6.2E-59
quartile effect	Reference	*P*=1.4E-02	*P*=2.1E-14	*P*=1.3E-36	
**Nomogram model**					
HRs (95% CI)	1.0	1.39 (0.860-2.25)	4.44 (2.94-6.71)	16.0 (10.8-23.7)	*P*=9.2E-78
quartile effect	Reference	*P*=1.8E-01	*P*=1.3E-12	*P*=8.9E-44	

Table S6. Univariate and multivariate Cox proportional hazard regression analysis of TACSs in the training, internal validation, external validation, and combined cohorts.

Training	Univariate analysis				Multivariate analysis			
	HR	(95%CI)		*P* Value	HR	(95%CI)		*P* Value
TACS1	0.168	0.088	0.323	<0.0001	0.153	0.072	0.325	<0.0001
TACS2	0.887	0.457	1.723	0.724				
TACS3	3.775	1.030	13.834	0.045	9.409	2.153	41.116	0.003
TACS4	0.179	0.108	0.297	<0.0001	0.231	0.122	0.434	<0.0001
TACS5	3.261	1.776	5.990	<0.0001				
TACS6	13.210	8.296	21.034	<0.0001	3.585	1.872	6.865	<0.0001
TACS7	0.535	0.278	1.032	0.062				
TACS8	6.629	2.724	16.133	<0.0001				
Internal validation								
TACS1	0.285	0.133	0.612	0.001	0.261	0.109	0.627	0.003
TACS2	1.069	0.410	2.789	0.891				
TACS3	12.633	2.735	58.362	0.001				
TACS4	0.088	0.043	0.179	<0.0001	0.135	0.058	0.315	<0.0001
TACS5	4.829	2.181	10.693	<0.0001				
TACS6	14.655	8.120	26.449	<0.0001	3.658	1.619	8.265	0.002
TACS7	0.525	0.206	1.337	0.177				
TACS8	4.689	1.676	13.117	0.003	3.738	1.226	11.395	0.020
External validation								
TACS1	0.072	0.019	0.268	<0.0001	0.133	0.033	0.539	0.005
TACS2	0.006	0.00001	3.984	0.123				
TACS3	5.234	0.148	184.567	0.363				
TACS4	0.181	0.101	0.324	<0.0001	0.383	0.170	0.866	0.021
TACS5	1.828	0.843	3.962	0.126				
TACS6	11.764	6.531	21.191	<0.0001	3.940	1.619	9.585	0.003
TACS7	0.040	0.002	0.931	0.045				
TACS8	12.028	3.929	36.823	<0.0001	5.323	1.470	19.273	0.011
Total								
TACS1	0.186	0.118	0.294	<0.0001	0.192	0.114	0.324	<0.0001
TACS2	0.863	0.508	1.465	0.585				
TACS3	5.729	2.281	14.392	<0.0001	5.924	1.983	17.693	0.001
TACS4	0.158	0.113	0.220	<0.0001	0.245	0.160	0.375	<0.0001
TACS5	3.013	2.002	4.535	<0.0001				
TACS6	12.963	9.517	17.657	<0.0001	3.556	2.293	5.516	<0.0001
TACS7	0.476	0.283	0.799	0.005				
TACS8	6.487	3.652	11.525	<0.0001	3.352	1.781	6.309	<0.0001

Table S7. 5-year disease-free survival prognosis of 995 breast cancer patients by single- and multi-prognosticator models.

Models	Low-risk	HR	95%CI	*P* value	AUC	Sensitivity	Specificity	Accuracy
TACS1	320 (32.2%)	2.22	1.73-2.86	3.6E-10	0.634	0.804	0.392	0.540
TACS2	251 (25.2%)	1.12	0.89-1.42	0.34	0.513	0.768	0.263	0.444
TACS3	904 (90.9%)	1.44	1.06-1.95	0.02	0.515	0.115	0.922	0.632
TACS4	387 (38.9%)	3.41	2.64-4.40	3.0E-21	0.721	0.829	0.511	0.625
TACS5	598 (60.1%)	1.96	1.60-2.40	4.6E-11	0.610	0.535	0.677	0.626
TACS6	543 (54.6%)	4.32	3.46-5.41	9.4E-38	0.771	0.742	0.707	0.720
TACS7	210 (21.1%)	1.43	1.09-1.87	0.01	0.546	0.840	0.240	0.455
TACS8	764 (76.8%)	1.66	1.33-2.06	5.3E-06	0.573	0.317	0.815	0.636
Clinical model (See text)	699 (70.3%)	3.30	2.70-4.03	2.9E-31	0.731	0.513	0.823	0.712
TACS1-3 (Old model)*	292 (29.3%)	2.58	1.97-3.39	8.3E-12	0.648	0.843	0.370	0.540
TACS4,6,8 (New model)*	599 (60.2%)	4.61	3.72-5.71	3.6E-44	0.802	0.700	0.771	0.746
TACS1-8 (Full model)	561 (56.4%)	6.15	4.86-7.77	3.6E-52	0.827	0.782	0.757	0.765

*TACS1-3 or TACS4, 6, 8 model is based on the same Ridge regression method of the TACS1-8 (full model).
